# Decolorization with Warmth–Coolness Adjustment in an Opponent and Complementary Color System

**DOI:** 10.3390/jimaging11060199

**Published:** 2025-06-18

**Authors:** Oscar Sanchez-Cesteros, Mariano Rincon

**Affiliations:** Department of Artificial Intelligence, Universidad Nacional de Educación a Distancia (UNED), Juan del Rosal, 16, 28040 Madrid, Spain; mrincon@dia.uned.es

**Keywords:** decolorization, color2gray, color warmth–coolness adjustment

## Abstract

Creating grayscale images from a color reality has been an inherent human practice since ancient times, but it became a technological challenge with the advent of the first black-and-white televisions and digital image processing. Decolorization is a process that projects visual information from a three-dimensional feature space to a one-dimensional space, thus reducing the dimensionality of the image while minimizing the loss of information. To achieve this, various strategies have been developed, including the application of color channel weights and the analysis of local and global image contrast, but there is no universal solution. In this paper, we propose a bio-inspired approach that combines findings from neuroscience on the architecture of the visual system and color coding with evidence from studies in the psychology of art. The goal is to simplify the decolorization process and facilitate its control through color-related concepts that are easily understandable to humans. This new method organizes colors in a scale that links activity on the retina with a system of opponent and complementary channels, thus allowing the adjustment of the perception of warmth and coolness in the image. The results show an improvement in chromatic contrast, especially in the warmth and coolness categories, as well as an enhanced ability to preserve subtle contrasts, outperforming other approaches in the Ishihara test used in color blindness detection. In addition, the method offers a computational advantage by reducing the process through direct pixel-level operation.

## 1. Introduction

Converting color images to grayscale presents a surprisingly complex challenge in computer vision. While seemingly intuitive for humans —we readily create black ink drawings of landscapes while preserving contrast between elements like a blue sky and green forests— achieving optimal contrast preservation across diverse image content remains an open problem [1]. The creation of grayscale images has a long history, dating back to the earliest human attempts at representation —drawing with charcoal on walls. However, this seemingly simple task became a technological challenge with the advent of early black-and-white televisions and, subsequently, computer image processing and computer vision.

The historical development of decolorization techniques reveals that significant advancements were not realized until the mid-first decade of the 21st century, largely due to the incorporation of methodologies for evaluating results [1]. Consequently, the most substantial progress occurred between 2004 and 2014. However, with the rise of deep learning and its inherent complexity in applying it to this problem, progress decelerated until renewed interest emerged recently. Currently, two primary categories of methods exist: those that apply weights to color channels and those that analyze contrast across areas —both locally and globally— resulting in varying outcomes and computational efficiency. Ultimately, the core objective is to develop a strategy for maintaining color contrast represented in three channels within a single grayscale channel.

This paper introduces a novel method for converting color images to grayscale that leverages principles of opponent color theory and warmth-coolness adjustments to preserve contrast. Unlike previous approaches that rely on weighted channel combinations or global contrast analysis, our Decolorization with Warmth–Coolness Adjustment (DWCA) method utilizes an Opponent and Complementary Color (OCC) system inspired by the anatomy of the retina and established perceptual color frameworks.

We first investigated both the anatomy of the retina and established color theories to relate neuronal activity within the retina to a color scale. From this relationship, we derived a straightforward transformation based solely on pixel information that yields a scale suitable for decolorization. Secondly, we examined the so-called color categories of warmth and coolness and their relation to this color system, establishing an operation where the warm–cool relationship is evaluated; subsequently, warm colors are amplified, and cool colors are reduced.

This article is organized as follows: First, we present a review of color representation and decolorization techniques and commonly used color contrast evaluation methodologies. Second, we detail our OCC system and DWCA method. Third, we evaluate the performance of our decolorization process and compare it to other representative methods. Fourth, we discuss the results obtained. Finally, we present conclusions and outline directions for future work.

## 2. Related Works

### 2.1. Color Representation in the Retina

Knowledge about how color is represented in the retina has advanced significantly since the 19th century, elucidating both its anatomical structure and functional mechanisms. The prevailing consensus indicates that the retina converts a trichromatic signal (derived from light) into a system of opponent color processes. Anatomically, photoreceptor cells (rods and cones) capture light signals: long (L), medium (M), and short (S) wavelengths, which are then processed by ganglion cells. Horizontal neurons within the receptive field modulate these signals [2]. Two primary types of ganglion cells exist: magnocellular cells with large receptive fields that process L+M signals; and parvocellular cells with smaller receptive fields that process L and M signals, alongside koniocellular cells, which handle S signals. Furthermore, the signal is processed through two parallel pathways: ON and OFF, as described by Schiller et al. [3] and Hubel [4], among others. The ON pathway represents stimulus intensity, while the OFF pathway reflects its absence. Consequently, the trichromatic information captured in the retina is transformed into a structure of two opposing pathways (ON and OFF), with four channels designated as L, M, S, and LM (for ON) and -L, -M, -S, and -LM (for OFF). White would represent maximum intensity across all channels (L, M, S, and LM) in the ON pathway and none in the OFF pathway; black would be the opposite.

### 2.2. Color Representation Systems

Human curiosity surrounding color has existed throughout history. Plato, in *Menon* (76d4-5) and *Timaeus* (67c3-68d7), and Aristotle, in *On Sense and Sensible Things* and *On Colors*, studied its properties and developed concepts such as visual flow within the perception system or that all colors originate from a mixture of four. Modern theories began with Newton and his *Opticks*, describing the color spectrum [5]. Subsequently, Thomas Young established the foundations of trichromatic theory [6], and in the mid-19th century, Von Helmholtz [7] defined the complete trichromatic color system (RGB). Goethe presented his theory of color in 1810 [8], indicating that visual perception plays an active role. A young Arthur Schopenhauer analyzed this idea and decided to elaborate a more advanced theory in 1816 [9], relating retinal activity to colors using a double division, one qualitative (through the relationship of opponent colors) and another quantitative (through the intensity of the activity). Hering described the opponent process theory based on Goethe’s studies and indirectly on Schopenhauer’s [10]. Munsell developed a system [11] based on Runge’s color sphere [12], where each color is identified through its properties of hue, lightness, and saturation. The HSV model, developed by Alvy Ray Smith in the 1970s to facilitate digital image processing, focuses on color components and is particularly useful for selecting hue, where V corresponds to color intensity. On the other hand, the HSL or HSI model, also centered on hue, represents it in a way that closely aligns with human visual perception. The CMYK subtractive color system arose from the need for color printing; concurrently, color theories within the field of art, developed by Itten [13] and Klee [14], among others, explored relationships between colors and their combinations. The 20th century saw significant advancements in color science, particularly with the establishment of the International Commission on Illumination (CIE) in 1931. The CIE developed standardized color spaces, such as CIE XYZ and CIE LAB, which provided a framework for quantifying and communicating color. The CIE LAB color space, introduced in 1976, was designed to be perceptually uniform, meaning that equal distances in the color space correspond to equal perceptual differences [15]. In recent years, the study of color has continued to evolve with the development of digital technologies. The RGB and CMYK color models have become essential for digital displays and printing, respectively. Additionally, advancements in color difference formulas, such as CIEDE2000, have improved the accuracy of color measurement and quality control in various industries [16].

The relationship of color with the system of visual perception is close; therefore, the trichromatic color vision theory is designed to represent colors in a way that closely aligns with how humans perceive differences and similarities between colors based on the physical properties of light, specifically wavelengths. Like the retina, it defines three ranges of frequency: long (L), medium (M), and short (S). While Newton’s theory posited that colors arise from the decomposition of light [8], Goethe and, notably, Schopenhauer [9] challenged this perspective. He conducted experiments on visual perception, emphasizing afterimages as evidence for an opponent color system processed at a higher level than mere physical effects of light. Schopenhauer elaborated upon these ideas with two core concepts:

- Retinal activity and color processing: he argued that retinal activity is intrinsically linked to color perception, countering the prevailing theory that colors are solely generated by light decomposition;
- Qualitative and quantitative division of activity: he further proposed that retinal activity can be divided both qualitatively and quantitatively.

The relationship between retinal activity and color processing, along with the qualitative and quantitative distinctions within that activity, suggests an underlying structure well-suited for analyzing opponent colors. White represents full retinal activity, black inactivity (or zero activity), and other colors exist on a scale between these extremes, establishing a quantitative division. Simultaneously, the concepts of activity and inactivity define an opponent relationship; they represent opposing poles that, together, encompass all retinal activity. In essence, each color can be characterized by both its degree of activity and its degree of inactivity.

### 2.3. Color Contrast

Contrast inherently implies difference, which suggests order and distance. It is a fundamental tool in image analysis to establish hierarchical and opposing relationships between image regions [17]. Empirical evidence suggests a natural connection between objective color-ordering principles and human perception [18]. However, understanding how humans categorize the visible spectrum remains a core challenge in cognitive science [19], and Euclidean distances within common color spaces often fail to accurately reflect perceived differences between colors [20].

**Figure 1:**
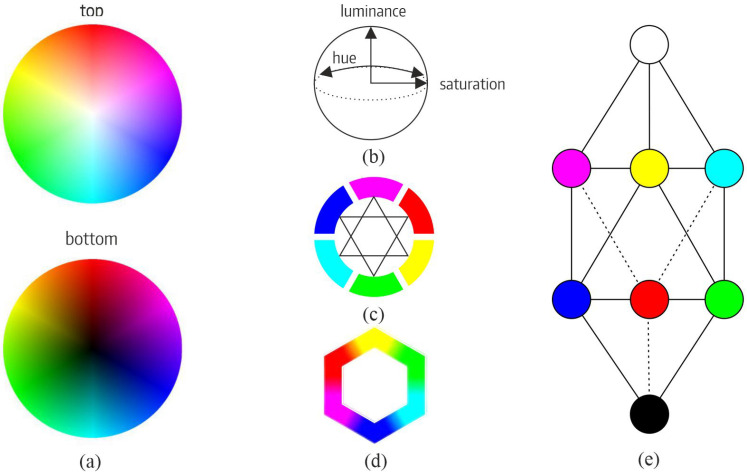
Examples of color representation systems based on perception. Note: (**a**) Runge’s color sphere; (**b**) HSL sphere; (**c**) Goethe’s and Hölzel’s complementary color wheel, which features a trichromatic system with red, green, and blue as the primary colors, and yellow, cyan, and magenta as the resulting secondaries; (**d**) Kueppers’ hexagon; and (**e**) Kueppers’ rhombohedron.

Establishing a scale among colors is essential for processing visual information and defining relationships of order, opponency, and complementarity. Historically, various models have attempted to represent these relationships. Munsell’s sphere [11] (see Figure 1a,b) organizes colors based on their position relative to neighboring hues and antipodes. Color wheels, popularized by Itten and Hölzel [13] (see Figure 1c), provide another common representation. Other approaches include Klee’s elementary stars and double pyramids [14], Kueppers’ hexagonal arrangement, where colors are related through their vertices [21] (see Figure 1d), and Kueppers’ rhombohedral scale, with yellow, cyan, and magenta at the apex, green, red, and blue below, and white and black representing the extremes (see Figure 1e).

The terms “opponent” and “complementary” colors have been used interchangeably in the past, leading to potential confusion. Recent research is revisiting these distinctions, as explored by Manzotti [22], Zeki et al. [23], and Pridmore [24]. Complementary colors are readily defined within the trichromatic system-pairs that, when combined, produce white (maximum values across RGB channels). However, identifying opponent colors is less straightforward.

Beyond their physical properties, colors also represent cognitive categories, establishing semantic relationships at varying levels of abstraction. Basic categories include “warm”, “cool”, “light”, and “dark”, with more specific distinctions like “light-warm” and “dark-cool” [25,26]. At higher levels, colors can evoke complex concepts, such as green representing “hope” or red signifying “passion” [17].

Berlin and Kay’s cross-cultural linguistic study [26] demonstrated the universality of color categories, largely independent of cultural influences, identifying 16 basic color terms. Rosch’s work [27], involving the Dani tribe in New Guinea, who initially categorized colors into only two groups: light-warm and cool-dark, revealed fascinating insights. Subsequent experiments with this group showed they learned primary colors (red, green, blue, and yellow –crucial for opponent color processing) more rapidly than individuals from other cultures. This suggests that color categories are established perceptually, much like the categorization of primary colors.

### 2.4. Decolorization Methods

**Figure 2:**
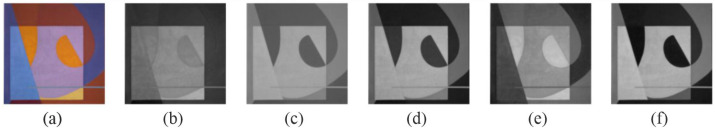
Examples Note: (**a**) image, (**b**) BT.601 recommendation (ITU), (**c**) Principal Component Analysis (PCA), (**d**) Grundland and Dodgson [32], (**e**) Kim et al. [33] and (**f**) Lu et al. [34].

Numerous decolorization methods have been developed over the years, employing diverse approaches and strategies with varying results; a universal solution remains elusive (Figure 2). These methods can broadly be categorized into several groups.

First, many techniques rely on weighting individual color channels to estimate their contribution to perceived luminance. These approaches often leverage established color spaces, like CIECAM97, L*a*b*, XYZ lum, and YCrCb Lum, and are reflected in various patents [28,29]. Furthermore, methods like those of Majewicz and Smith [30] and NG et al. [31] calculate channel differences based on principles of human visual perception. A particularly prevalent standard in computer applications is the ITU Recommendation BT.601, which is frequently utilized within common computer vision libraries like Matlab and Photoshop.

Second, some approaches directly analyze color channels to reduce dimensionality or identify visually salient features. For example, Seo and Kim [35] employed Principal Component Analysis (PCA). Others utilize channels representing visual properties, such as intensity, hue, texture, and pictorial effects, often applying thresholds to these components [36].

Third, a significant category of methods assesses contrast within image regions to determine pixel relevance for grayscale conversion. This analysis can be local, global, or a combination of both. The goal is to estimate each pixel’s contribution to the final grayscale scale. These techniques typically involve algorithms operating on individual pixels or groups of pixels. Examples include the following:

- Local approaches: Bala et al. [37] improved contrast between adjacent colors; Neumann et al. [38] selected color gradients locally for 2D integration; Lu et al. [39] preserved existing local contrast; and Zhang and Liu [40] enhanced Perceptual Group Difference (PGD).
- Global approaches (linear): Gooch et al. [41] utilized chromaticity and luminosity changes, and Grundland and Dodgson [32] added a fixed amount of chroma and intensity.
- Global approaches (nonlinear): Kim et al. [33] processed luminance, chroma, and tone; Ancuti et al. [42] incorporated three RGB channels and an additional image to preserve color contrast; Ancuti et al. [43] mixed saturation and hue channels for salience preservation; Liu et al. [44] employed gradient correlation with a nonlinear global mapping in the RGB space; and Song et al. [45] used a probabilistic graphical model to minimize an integral, preserving visual key elements.
- Local and global approaches: Smith et al. [46] globally assigned gray values while locally improving contrast; and Kuk et al. [47] encoded local and global contrast within an energy function using a target gradient field.

Finally, recent advancements leverage machine learning techniques. Lin et al. [48] trained partial differential equations (PDEs). Hou et al. [49] utilized deep learning, specifically Deep Feature Consistent Deep Image Transformation (DFC-DIT), which employs a CNN as a nonlinear mapper to transform images while maintaining deep feature consistency through another pretrained and fixed CNN. Liu and Zhang [50] combined local and global features within a CNN framework, recognizing the critical role of exposure in human visual perception. Cai et al. [51] employed deep representations to extract content-based information aligned with human visual perception, automatically selecting an appropriate grayscale scale for image decolorization.

### 2.5. Evaluation of Color Contrast in the Decolorization

Evaluating decolorization methods presents a significant challenge, requiring both quantitative and qualitative assessment. Čadík [52] conducted an early comparative study using subjective evaluation. In 2008, he performed two experiments to assess user preference: one with the original color image as a reference and another without any reference. The study utilized the Cadik dataset, comprising 24 images across diverse themes, and involved 121 observers (men and women aged 18–41 years with normal vision) who made a total of 20,328 observations. The results indicated that the Decolorize converter by Grundland and Dodgson [32] and Smith et al. [46] received the highest rankings, while the method of Bala et al. [37] performed comparatively worse.

However, a need arose for a more quantitative evaluation methodology. Lu et al. [39] introduced the Color Contrast Preserving Ratio (*CCPR*), designed to assess whether contrast is maintained between image regions after decolorization above a defined threshold:

(1) $$CCPR=\frac{\#(x,y)|(x,y)\;\in \Omega ,|gx-gy|\geq \tau }{\left||\Omega |\right|}$$

Here, (x,y) represents a pixel pair, with $g_{x}$ and $g_{y}$ denoting the resulting grayscale values after decolorization. Ω is the set of pixel pairs where |x−y|≥τ. ||Ω|| denotes a sample of randomly selected pixel pairs across the image. #(x,y)|(x,y)∈Ω,|gx−gy|≥τ represents the count of pixels within Ω where the contrast remains after decolorization. *CCPR* yields a value between 0 and 1, with 1 indicating complete color preservation.

Lu et al. initially applied *CCPR* to the Cadik dataset using τ>4, which is considered discernible contrast by human perception. Subsequently, in 2014, they refined their evaluation methodology [34], introducing a larger dataset, Color250, and the color fidelity ratio, *CCFR*:

(2) $$\begin{array}{cc} CCFR & =1-\frac{\#(x,y)|(x,y)\in \Theta ,|x-y|\leq \tau }{\left||\Theta |\right|} \end{array}$$

where Θ is the set of pixel pairs where $|g_{x}-g_{y}|>\tau$. This quantifies the number of pixel pairs exhibiting high contrast after decolorization but falling below the original color threshold, i.e., |x−y|≤τ. In essence, it measures unexpected increases in color contrast during the decolorization process.

To combine these two ratios, they introduced the E-score as their harmonic mean:

(3) $$E-score=\frac{2\cdot CCPR\cdot CCFR}{CCPR+CCFR}$$

They validated this evaluation methodology through a human study, demonstrating a correlation between E-scores and subjective preferences.

In 2015, Ma et al. proposed the C2G-SSIM (structural similarity index) [53], which quantifies structural information preservation. The ratio calculates the mean and standard deviation for each component, adjusting their relative importance using the following equation:

(4) $$q\left(x_{c}\right)=L{\left(x_{c}\right)}^{\alpha }\cdot C{\left(x_{c}\right)}^{\beta }\cdot S{\left(x_{c}\right)}^{\gamma }$$

Here, $L{\left(x_{c}\right)}^{\alpha }$ represents the luminance component, $C{\left(x_{c}\right)}^{\beta }$ the color contrast, and $S{\left(x_{c}\right)}^{\gamma }$ the structure. $x_{c}$ denotes the spatial coordinate acting as a center for the SSIM calculation; α, β, and γ are weighting parameters used to prioritize each component. The specific equations for calculating each component’s C2G-SSIM value are detailed in their original publication.

Finally, in 2023, Ayunts and Agaian introduced TIA and WTIA ratios [54] designed to evaluate the preservation of relevant regions after decolorization and the maintenance of the original image structure (color, shape, texture, etc.).

While current metrics focusing on color contrast preservation and structural maintenance offer valuable quantitative insights, they inherently lack the capacity to assess alterations in visual meaning; consequently, researchers often employ qualitative evaluations using images containing inherent ambiguities to compare results across methods and verify their accuracy.

## 3. Methods

### 3.1. Opponent and Complementary Color System (OCC)

In this section, we describe our Decolorization with Warmth–Coolness Adjustment (DWCA) method. Our approach leverages a bio-inspired color representation system based on opponent and complementary colors, which we term OCC (Opponent and Complementary Color). This system facilitates the identification of these relationships, enabling a pixel-by-pixel decolorization process that incorporates warmth or coolness adjustments.

#### 3.1.1. Relationship Between Color and Retinal Neural Activity

**Figure 3:**
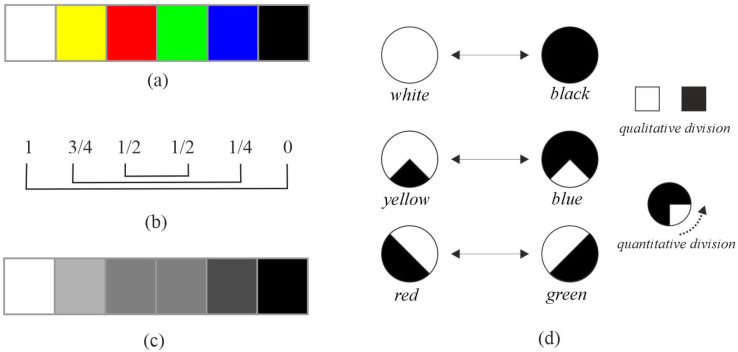
Color scale based on the divided activity of the retina. Note: (**a**) primary colors; (**b**) color scale based on divided retinal activity; (**c**) grayscale; and (**d**) relationship between the color scale and divided activity. In (**d**), the white sections represent activity, and the black sections represent inactivity (qualitative division); the area size represents the level of each (quantitative division).

The retina processes visual information through a pathway and parallel architecture. Initially, cone photoreceptors capture light in a trichromatic (RGB) system. Subsequent neural structures transform this into a four-channel system (L, M, S, and LM). Following Schopenhauer’s concept of divided retinal activity (activity versus inactivity), we define ON and OFF pathways as being opponent such that their combined activity represents complete activity. Consequently, based on this theory, each color can be associated with a degree of both retinal activity and inactivity. For example, yellow might represent 3/4 activity and 1/4 inactivity. In Figure 3, we relate Schopenhauer’s proposed color scale to retinal activity for primary colors. The figure establishes opponent relationships between yellow and blue, red and green, and white and black (Figure 3b). The sum of the values across these pairs represents full activity (Figure 3d). This color gradation relative to retinal activity allows for opposing relationships; for instance, red exhibits 1/2 activity in the ON pathway and 1/2 in the OFF pathway, while its opponent, green, displays the inverse relationship (see the correspondence between Figure 3b,c).

**Figure 4:**
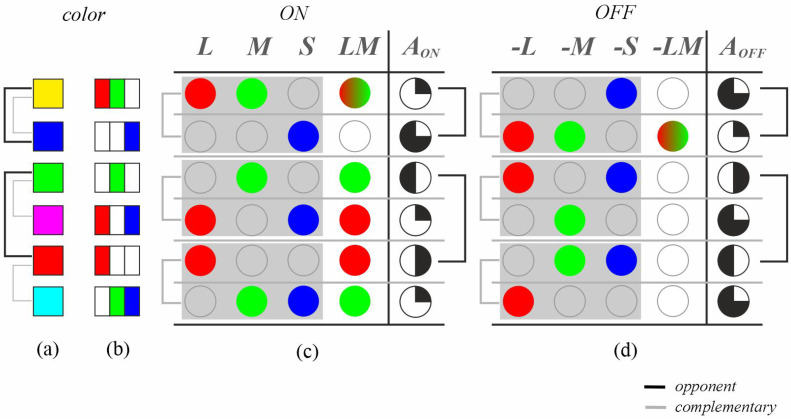
Opponent and complementary relationships of the OCC system. Note: (**a**) primary colors; (**b**) RGB channels; (**c**) OCC channels in the ON path; and (**d**) OCC channels in the OFF path. To represent OCC channels, the L channel uses red, M uses green, S uses blue, and LM uses a combination of red + green.

To quantify this relationship, we transform the four channels $c_{ON}=\left\{L,M,S,LM\right\}$ into the ON pathway and $c_{OFF}=\left\{-L,-M,-S,-LM\right\}$ into the OFF pathway to derive a “neural activity” value. Consistent with Schopenhauer’s concept of divided retinal activity, we define opponent ON and OFF pathways carrying signals representing activity and inactivity, the sum of which represents “full activity” (FA). We assume each channel signal is within the range [0,1] and that full activity is equal to 1. The simplest estimation of activity and inactivity in the ON and OFF pathways assumes homogeneity across channels, assigning them equal weight. Therefore, we define activity $A_{ON}$ and inactivity $A_{OFF}$ as follows:

(5) $$A_{ON}=\frac{L+M+S+LM}{4}$$

where LM=min(L+M,1), and

(6) $$A_{OFF}=\frac{-L+-M+-S+-LM}{4}\;$$

where -LM=1−LM. This transformation aligns with Schopenhauer’s concept of divided activity (both qualitatively and quantitatively) and relates to Hering’s opponent color process theory through the combinations with channels L, M, S, and LM. Figure 4 illustrates the activity/inactivity values for primary and secondary colors (Figure 4a), demonstrating the opponent relationships derived from Equations (5) and (6). The theory of opponent colors establishes relationships between yellow-blue, red-green, and white-black, which are generalized using ON/OFF activity pathways (Figure 4c,d). Specifically, the opponent color has an activity of $1-A_{ON}$ and an inactivity of $1-A_{OFF}$. Furthermore, relationships between yellow-blue, red-cyan, and green-magenta are complementary because the sum of their L, M, and S channel values equals full activity (white), as shown in Figure 4b (representing each color’s RGB channels with active and inactive components). Thus, opponent processes relate to retinal activity and inactivity, while complementary relationships reflect signals captured by the retina. This color system, derived from Schopenhauer’s theory and its relationship to separate and parallel retinal pathways, is termed OCC (Opponent Complementary Color), enabling a description of both relationships and providing values for each case; therefore, as a color system, it positions each color on a scale representing its neural activity.

**Figure 5:**
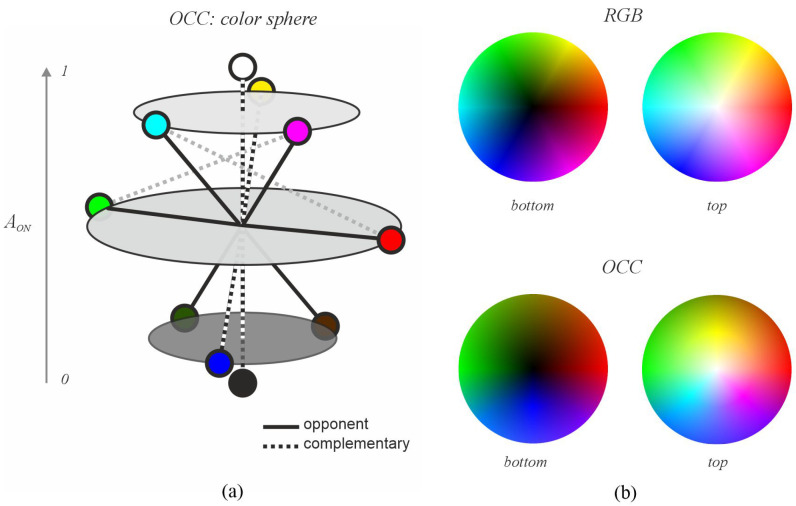
OCC color sphere. Note: (**a**) primary and secondary colors, with their opponents and complementaries in the OCC color sphere; and (**b**) comparison between OCC and RGB.

The OCC can be represented as a sphere where the vertical axis represents activity in the ON pathway ($A_{ON}$), the azimuthal angle represents hue distributed around the sphere according to the position of primary colors, and the radius represents saturation. Figure 5a schematically illustrates how the OCC system organizes colors to establish a scale facilitating analysis of opposing colors. It demonstrates that for any given color, its opposite can be determined as its antipode. For example, a color with an OCC representation (L=1, M=0.5, S=0.5, and LM=1) is located at $A_{ON}=3/4$, on the 45° parallel in the upper hemisphere and the 0° meridian (red). Its opponent has characteristics of $A_{ON}=1/4$ on the 45° parallel in the lower hemisphere and the 180° meridian (green), corresponding to (L=0, M=0.5, S=0, and LM=0.5) in the OCC system.

Figure 5b compares color distributions in RGB and OCC using primary and secondary colors. Hue is distributed at equally spaced angles among the primary colors (three in RGB with 120° separation and four in OCC with 90° separation), starting by assigning red to 0°. In RGB, the vertical axis is governed by luminance, which places primary and secondary colors on the equator of the sphere (luminance = 0.5) and trends towards white (upper hemisphere pole) or black (lower hemisphere pole). In contrast, in the OCC system, colors are positioned according to their activity values: yellow, cyan, and magenta reside in the upper hemisphere ($A_{ON}=0.75$), red and green are on the equator (as in RGB, $A_{ON}=0.5$), and blue is in the lower hemisphere ($A_{ON}=0.25$). Other colors are positioned accordingly. In RGB, there’s a gap between red and green, green and blue, and blue and red. These gaps correspond to yellow, cyan, and magenta, respectively. The OCC system eliminates these gaps, creating a more continuous space. In RGB, luminance establishes a scale from black to white, while hue only assigns order relationships without any scale. However, the OCC system introduces a scale in hue and combines it with luminance, allowing for a more precise and realistic description of each color in relation to its attractiveness in perception, establishing a relationship between retinal activity, stimulus intensity, and attraction.

#### 3.1.2. Definition of Warm and Cool Categories

Colors are often perceived as subjective categories, establishing semantic relationships at various levels of abstraction, beginning with “warm” and “cool” [25,26]. While these categories result from human perception, and the association of a color with warmth or coolness is inherently subjective, we can establish a model based on how our visual system processes color information. This model leverages the ON pathway in the retina, which transmits signals related to color luminance and hue.

The ON pathway utilizes three primary channels: L (Long wavelength, roughly corresponding to red), M (Medium wavelength, roughly corresponding to green), and S (Short wavelength, roughly corresponding to blue). The relative strength of these channels contributes significantly to how we perceive a color’s warmth or coolness. Colors dominated by the L channel are perceived as warm, while those dominated by the M and S channels are perceived as cool.

To quantify this relationship, we define dominance using the following equation:

(7) D=L−MS

where MS=min(M+S,1). This formula calculates a “Dominance” score (D) by subtracting the combined strength of the M and S channels from the strength of the L channel. Warmth or coolness is then determined as follows:

(8) warm:if;D≥0

(9) cool:if;D<0

A positive Dominance score (D≥0) indicates that the L channel is more active than the combined M and S channels, classifying the color as warm. Conversely, a negative Dominance score (D<0) signifies greater activity in the M and S channels, leading to a classification of cool.

Table 1 summarizes these relationships. It is important to note that some colors exist near the boundary between warm and cool. For example, yellow-greenish hues have a significant presence of both M (green) and L (red) channels, while yellow-reddish hues exhibit similar characteristics but with a slightly stronger influence from the L channel. The precise categorization in these cases depends on the relative strength of each channel, as determined by Equation (7).

### 3.2. Decolorization with Warmth–Coolness Adjustment (DWCA)

#### 3.2.1. Converting Color to Grayscale

To effectively manage and manipulate color information during decolorization, we utilize the Opponent and Complementary Color (OCC) system, which offers a valuable advantage by representing colors on a scale where the perceptual attributes of hue, lightness, and saturation are inherently intertwined, facilitating a more holistic approach to color processing. This foundation in OCC, which relates color perception directly to retinal activity, informs our initial step: determining the position of each pixel on a grayscale scale based on its activity value, $A=A_{ON}$ (Equation (5)).

#### 3.2.2. Adjustment of Grayscale Position Based on Warmth or Coolness

While OCC provides a grayscale scale that generally reflects color perception, it can group warm (reddish) colors and cool (greenish) colors at similar intensity levels. Recognizing that the L channel dominates in warm colors and the M and S channels are more prominent in cool colors, we apply an adjustment to shift the grayscale position based on the *D*, warmth, or coolness. This is implemented using a sigmoid function over the interval [−1,1] to ensure even minor variations exert a significant influence. The sigmoid function is employed to prevent small values of *D*—where adjustments are most needed—from having minimal impact on the initial grayscale value *A*:

(10) $$A^{\prime }=A+(a*\left((\frac{1}{1+e^{-3D}}\right)-0.5))\;$$

Here, $A^{\prime }$ denotes the adjusted grayscale value, *A* is the initial activity value, and *a* is a coefficient that modulates the influence of warmth or coolness, determined by D:

(11) ifD≥0,a=Welsea=C

where *W* and *C* are configurable constants representing the strength of warmth and coolness adjustments, respectively. The exponent ‘3’ in the sigmoid controls the steepness of the curve, influencing how strongly these differences affect the adjustment.

**Figure 6:**
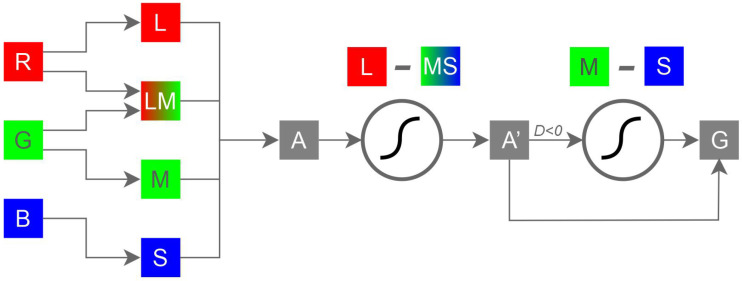
DWCA functional architecture.

To prevent the distinction between cool colors from being lost —for example, between green and blue— we further refine the adjustment for cool colors to emphasize channel *M* over channel *S*. This prioritization stems from the OCC scale where increased prominence of the M channel corresponds to colors occupying a higher position on the grayscale scale. This refinement is achieved through

(12) $$if\;D<0,\;G=A^{\prime }+(s*\left((\frac{1}{1+e^{-3(M-S)}}\right)-0.5))\;else\;G=A^{\prime }$$

where *s* is a coefficient, typically kept low (below 0.3) to prevent saturation effects. If *s* were too high, this operation would excessively amplify the green channel’s contribution, pushing it closer to its original value in *A* while simultaneously reducing the blue channel towards zero. The final grayscale value, *G*, represents the output of this decolorization process. Figure 6 illustrates the complete workflow, showing the relationships between RGB channels, the OCC system, and the warmth/coolness adjustment stages.

**Figure 7:**
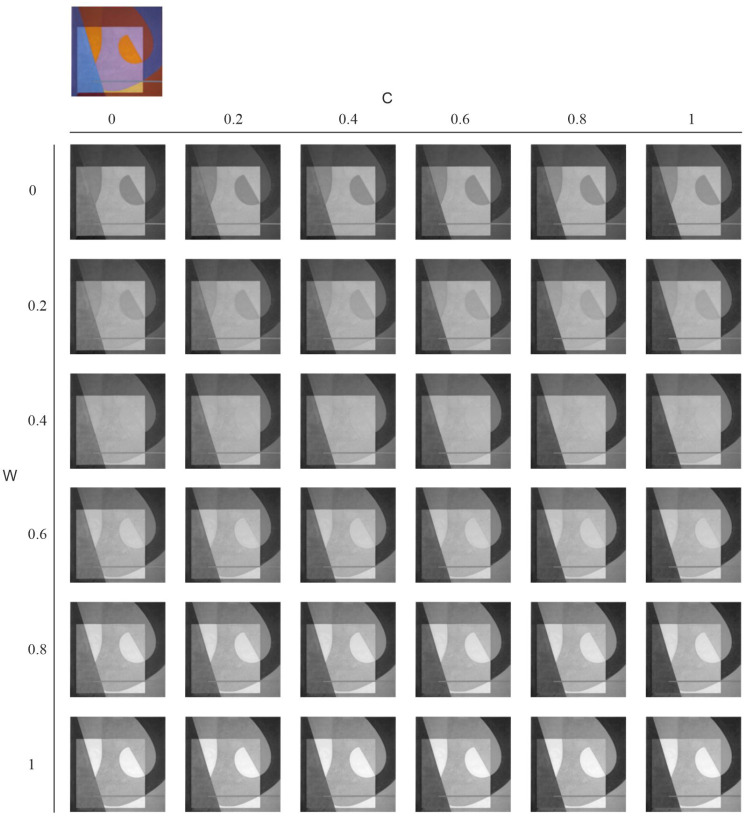
Example of DWCA with different combinations of W and C.

Figure 7 demonstrates the impact of varying *W* and *C*. We observe that parameter tuning significantly affects the outcome; for example, when W=0 and C=0, blue and orange areas exhibit similar grayscale values, effectively reducing color contrast. Increasing *W* brightens the orange regions, transitioning from dark tones at lower values (below 0.4) to a level matching the background gray at higher values (W=0.4). Conversely, increasing *C* diminishes the intensity of cool colors, particularly blues. A suitable configuration for this example appears to be W=0.6 and C=0.4.

## 4. Experimental Benchmarking

We evaluate our proposed method quantitatively and qualitatively against relevant decolorization techniques, categorized by their approach. We avoid subjective human evaluation, relying on established quantitative metrics: *CCPR*, *CCFR*, and CG2-SSIM. Furthermore, to assess performance in scenarios with limited color contrast —specifically, the ability to preserve subtle distinctions between reddish and greenish hues— we also incorporate the Ishihara test, commonly used for detecting color vision deficiencies. All methods were implemented using their original codebases, translated from MATLAB 8.3 to Python 3.9 for consistent evaluation across all techniques.

### 4.1. Datasets

**Figure 8:**
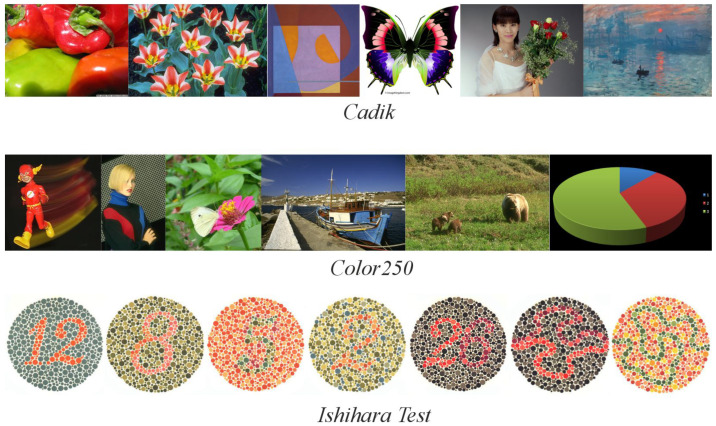
Examples of images from the Cadik and Color250 datasets, and plates from the Ishihara test.

The Cadik [52] dataset comprises 24 images, while Color250 [39] contains 250 images. Both datasets feature diverse content, including photographs and graphics, providing a comprehensive evaluation (examples are shown in Figure 8).

The Ishihara test [55] is a standard diagnostic tool for color vision deficiencies (CVDs). CVD is a genetically inherited condition affecting retinal receptors’ sensitivity to specific wavelengths, leading to difficulty distinguishing between hues, particularly red and green. In severe cases, blue may also be affected. This test is especially relevant because it presents images with low contrast between closely related colors, making it ideal for evaluating methods’ ability to preserve subtle color distinctions.

The test identifies various types of anomalies: protanomaly (reduced red detection), protanopia (absence of red detection), deuteranomaly (reduced green detection), and deuteranopia (absence of green detection).

The original Ishihara test consists of 38 plates (examples are shown in Figure 8), each containing textured circles with hidden numbers or lines. These elements are designed to be visible only to individuals with normal color vision, while those with specific CVDs will perceive them differently:

- Plates 1 and 38: Numbers should be clearly visible to all individuals with normal color vision.
- Plates 2–9: A number is visible to individuals with normal color vision but appears different or invisible to those with red or green deficiencies.
- Plates 10–17: A number is visible to individuals with normal color vision and is not visible to those with red or green deficiencies.
- Plates 18–21: Numbers are not visible to individuals with normal color vision but appear to those with red or green deficiencies.
- Plates 22–25: A two-digit number is visible to individuals with normal color vision; the first digit appears to those with red deficiencies and the second digit to those with green deficiencies.
- Plates 26 and 27: Lines are visible to individuals with normal color vision, with only the top portion visible to those with red deficiencies and the bottom portion to those with green deficiencies.
- Plates 28 and 29: Lines are visible only to individuals with red or green deficiencies.
- Plates 31–33: Numbers are visible only to individuals with normal color vision.
- Plates 34 and 35: Normal vision detects a green line, while those with red and green deficiencies detect a violet line.
- Plates 36 and 37: Normal vision detects an orange line, while those with red and green deficiencies detect a violet line.

### 4.2. Setup

To facilitate comparison, we selected representative methods from each category:

- Weighted channels: BT.601 recommendation (ITU);
- Channel analysis: Principal Component Analysis (PCA);
- Global linear contrast: Decolorization (D) with global contrast using a linear function to adjust chroma and intensity, as described by Grundland and Dodgson [32];
- Global nonlinear contrast: Nonlinear Global Map (NGM), derived from luminance, chroma, and hue functions, proposed by Kim et al. [33];
- Local contrast: Contrast Preserving Decolorization (CPD), employing a nonlinear real-time function to preserve local contrast, as detailed by Lu et al. [34].

#### 4.2.1. Parameter Configuration

For our proposed DWCA method, we explored two parameter configurations for W and C:

- Quantitative evaluation and Ishihara test: W=0.4, C=0.4, and s=0.3 (a fixed, low value). This configuration was chosen to minimize alterations to the original color palette while still achieving decolorization.
- Qualitative evaluation: The values of *W*, *C*, and *s* were adjusted on a per-image basis to optimize visual results. The default value for *s* was 0.3; when a different value was used, this is explicitly stated in the results section. We prioritized maintaining subtle color differences during this evaluation.

#### 4.2.2. Evaluation

For quantitative evaluation, we analyzed the average of *CCPR*, *CCFR*, and E-score for color contrast and C2G-SSIM for structural preservation. The Color250 dataset is used in both cases due to its larger number of images. *CCPR* and *CCFR* are calculated using a sample of 80 randomly selected pixel pairs from across the image, including neighboring and distant pixels, following the authors’ recommendations. Second, the Ishihara test is applied to check the low contrast between the red and green ranges. Additionally, a qualitative evaluation is performed using images from the Cadik dataset, as used in the article by Lue et al. [34], and a detailed analysis of Monet’s painting «Impression, Sunrise» is conducted due to its complex warm and cool contrasts.

## 5. Experimental Results

Detailed below are the findings from two complementary analyses –one quantitative and the other qualitative.

### 5.1. Quantitative Analysis

For the quantitative component, we analyzed performance using four metrics—*CCPR*, *CCFR*, E-score, and CG2-SSIM– on the Color250 dataset, in addition to the Ishihara test.

#### 5.1.1. Color Contrast

Table 2 presents the average results obtained at minimum contrast thresholds (τ) of 4, 10, and 20. CPD consistently outperformed other methods for *CCPR*, achieving scores of 0.88, 0.80, and 0.72 across these thresholds. DWCA and Method D demonstrated comparable performance (0.87, 0.78, and 0.66), while PCA exhibited the greatest variability, yielding scores of 0.81, 0.69, and 0.54.

Regarding *CCFR*, DWCA achieves lower values than other methods in τ=20, particularly BT.601, PCA, and CPD. At (τ=4 and τ=10), differences are minimal; all values remain close to 1. This trend impacts the E-score, where DWCA demonstrates slightly lower scores compared to D and especially CPD at minimum thresholds τ=20.

#### 5.1.2. Structural Preservation Analysis

Table 3 presents the average C2G-SSIM scores obtained by each method. BT.601 achieves the highest score (0.96), 0.03 higher than CPD and 0.05 higher than DWCA. The differences are more substantial when compared to other methods, including a difference of 0.07 with D, 0.22 with NGM, and 0.42 with PCA.

#### 5.1.3. Ishihara Test

**Figure 9:**
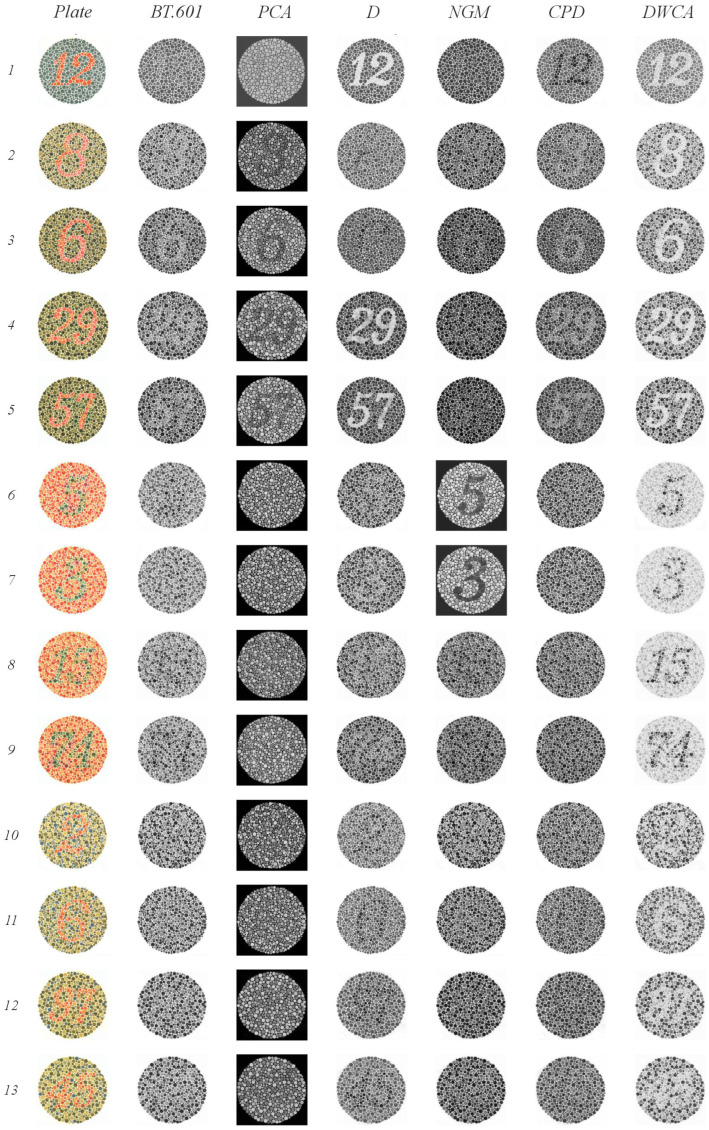
Results of the Ishihara test, plates 1–13.

**Figure 10:**
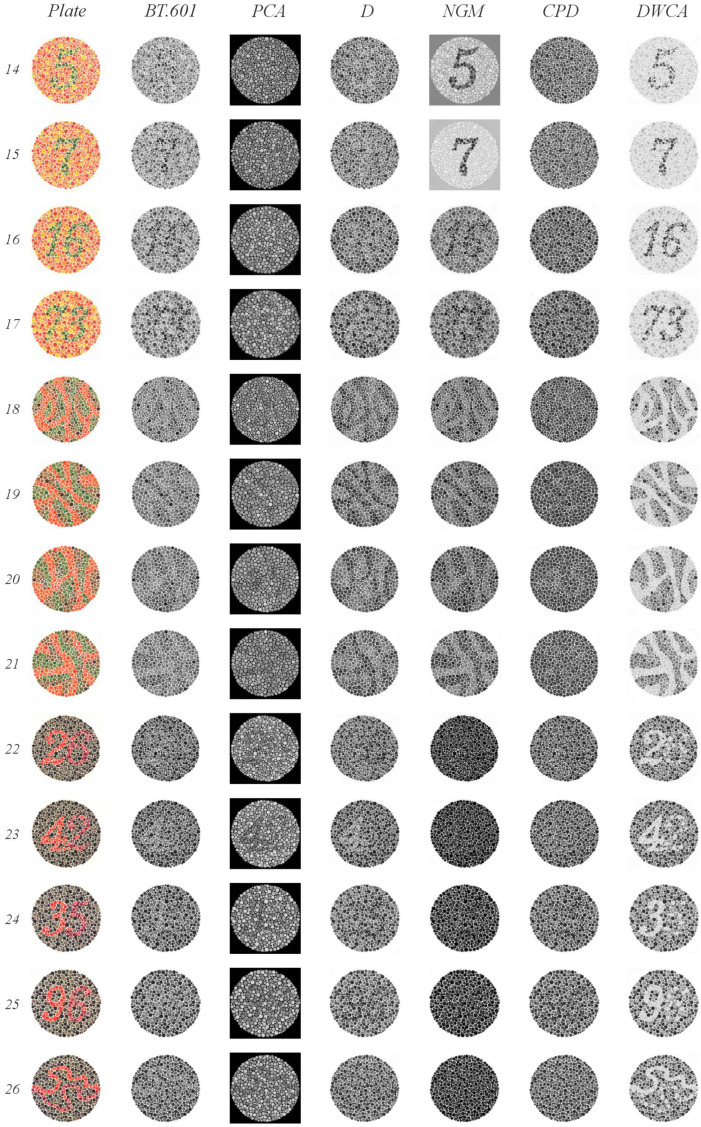
Results of the Ishihara test, plates 14–26.

**Figure 11:**
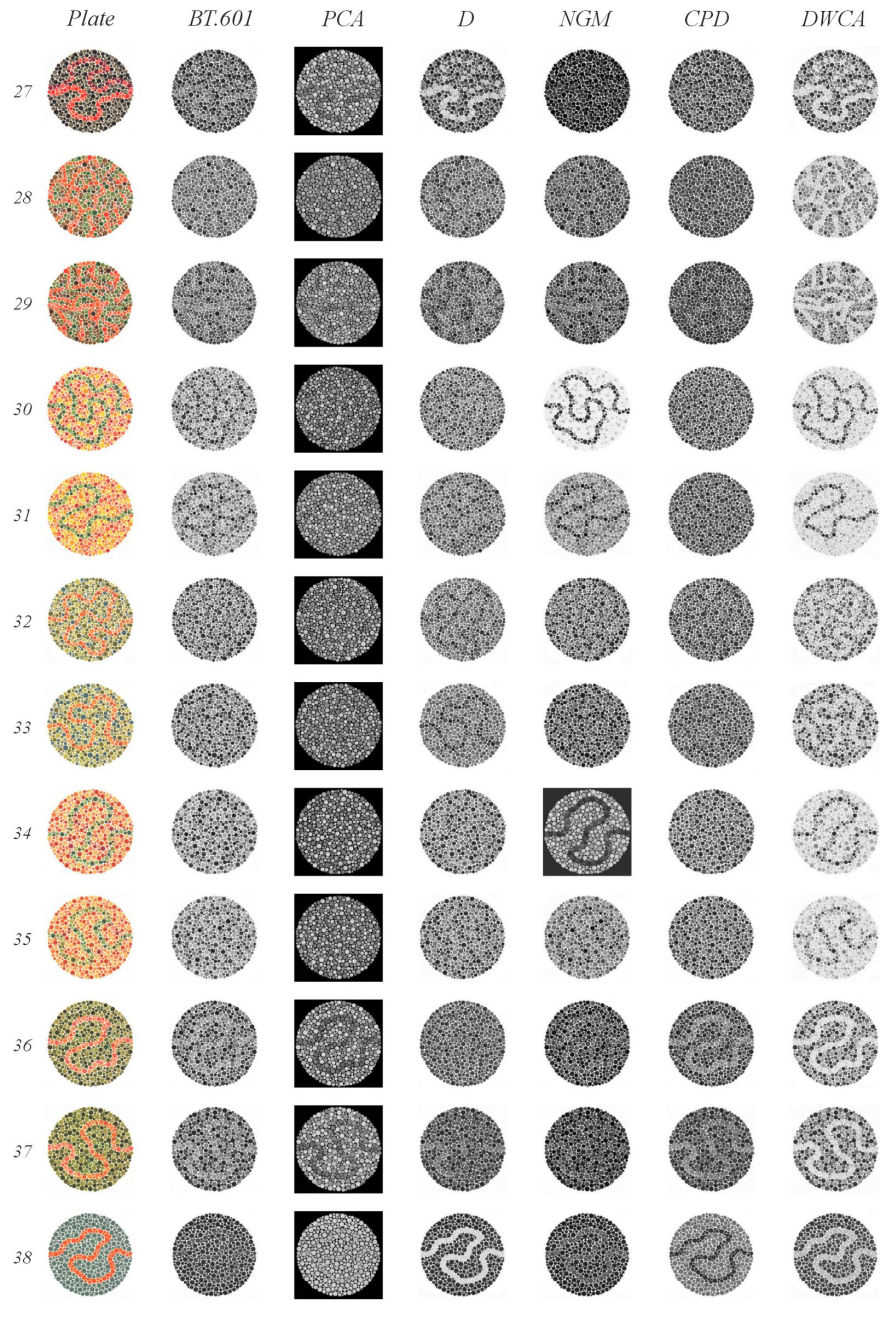
Results of the Ishihara test, plates 27–38.

Figure 9, Figure 10 and Figure 11 illustrate the results of our Ishihara test evaluation. Table 4 summarizes the accuracy percentages achieved by each algorithm across all plates. The following details provide a breakdown of performance for each method:

- BT.601: It detected four plates with orange figures on a green background (plates 2–5) and two, albeit faintly, with green figures on an orange background (plates 8 and 9). It also correctly identified three plates with green figures and orange backgrounds (plates 15, 16, and 17), as well as two lines (plates 30 and 31). The normal gaze line was successfully detected in plates 36 and 37.
- PCA: It detected four plates with orange figures on a green background (plates 2–5). Notably, it identified numbers in plates 22 through 25 that were only visible when red or green range detection failed. It struggled to detect lines in plates 26 and 27 when there were issues with green range detection; however, the line was detected correctly in plates 36 and 37.
- D: It detected plates 1 and 38 (expected to be visible by all methods) and two plates with orange figures on a green background (plates 4 and 5). In plates 18 through 21, it identified lines. One plate from the groups of 22 through 25 and 27 was detected. Plates 32 and 33, which had a green background, were correctly identified. Finally, despite some blurring, it successfully detected the orange line in plate 37 (normal vision), which featured a green background.
- NGM: It detected plates 4 and 5 (green figures on orange backgrounds); from groups 10–17, it detected 14 and 15 clearly, and plate 16 was slightly blurry. It identified the lines in plates 18–21. Plates 30 and 31 were detected within groups 30–33, and plate 34 was detected from blocks 34–35.
- GPD: It detected plates 1 and 38. From groups 2–9, it detected only the plates with a green background (plates 2–5). In plate 20, instead of detecting lines, it incorrectly displayed the number 45, which is typically only visible when red or green range detection fails. It also correctly identified plates 36 and 37.
- DWCA: Successfully detected all plates without any issues.

DWCA consistently achieved perfect detection across all plates. The other methods demonstrated limitations, particularly in detecting orange figures against a green background—most pronounced for CPD, PCA, and BT.601, with less difficulty observed for D and NGM.

### 5.2. Qualitative Analysis

**Figure 12:**
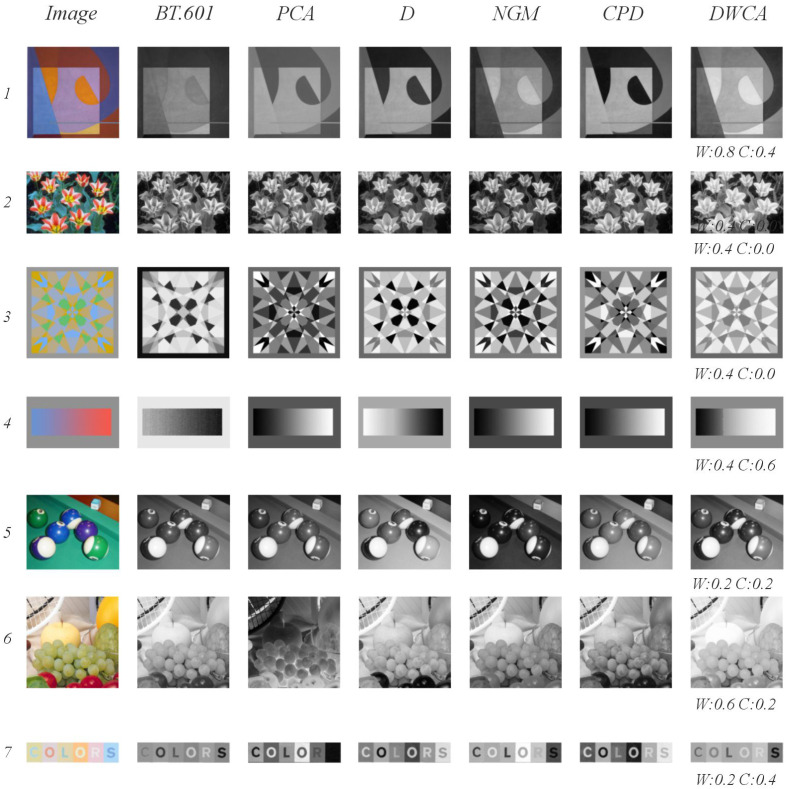
Qualitative examples of decolorization results on the Cadik dataset.

Figure 12 presents a series of illustrative examples demonstrating how color contrast challenges can impact feature preservation during decolorization. In Image 1, subtle intensity variations within color ranges pose difficulties for methods lacking local or global contrast analysis; BT.601 exemplifies this limitation. Conversely, approaches incorporating contrast analysis, such as D, NGM, and GPD, can enhance and even alter the perceived structure. This is particularly noticeable in the orange semicircle, where decolorization results vary significantly —ranging from darker to lighter shades. Notably, DWCA maintains a low overall contrast while effectively preserving intensity differences and the relationship between the figure (warm) and the background (cool). Image 2 presents a scenario with high saturation of white petals juxtaposed against magenta and yellow drawings, making decolorization challenging. The primary issue is the contrast between the white petal and the yellow; only DWCA achieves adequate contrast without significantly distorting the drawing’s shape. In Image 3, the ambiguity between the figure and background leads to variable outcomes —for example, the central cross, clearly visible in the color version, is discernible only with NGM and DWCA. Image 4 investigates the relationship between warm colors and lightness and cool colors and darkness; only BT.601 and D exhibit an inverse correlation. Image 5 features billiard balls with low hue and intensity, resulting in divergent decolorization outcomes. While D performs reasonably well, DWCA effectively differentiates green from blue balls even at a saturation value of s=0.5. Image 6 showcases lemons, golden apples, and grapes —all predominantly yellow— exhibiting high intensity and low contrast, making it difficult to maintain the original color scale; DWCA is the only method that preserves this scale effectively. Lastly, Image 7 exhibits very low contrast with high intensity, hindering the readability of the word “colors”. The results are highly variable: BT.601 homogenizes the background of the letters, reducing original contrast; PCA renders the letter “s” nearly invisible; D increases the contrast between the letters and their background; NGM makes the letters “r” and “s” difficult to distinguish from the background; CPD enhances contrast in the letters “c” and “o”. DWCA maintains a contrast closer to the original, preserving both the figure–background relationship and the internal structure of each letter, although the blue of the “s” appears slightly darker.

**Figure 13:**
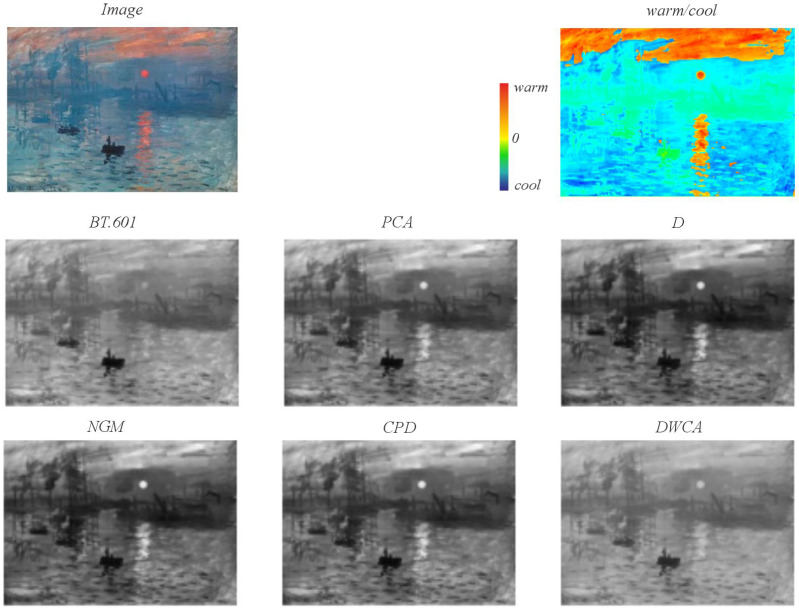
Decolorization of a painting: Claude Monet’s “Impression, Sunrise” (1872). Heatmap illustrating contrast values for warm and cool color regions.

Figure 13 illustrates the decolorization of Claude Monet’s iconic painting “Impression, Sunrise” (1872). This artwork presents a visual features challenge with low contrast within cool color ranges and higher contrast between warm and cool colors. The prevalence of blue as the dominant hue provides a valuable benchmark for evaluating each decolorization method’s performance. A heatmap displaying warm and cool regions is included to facilitate a visual comparison of the methods’ results. The primary color contrast resides in the sun and its reflection (warm ranges) on the water surface (cool ranges), where the aforementioned low-contrast issues are particularly evident. BT.601 exhibits minimal contrast between warm and cool regions, whereas PCA demonstrates a notably higher, albeit potentially artificial, contrast. D, NGM, and CPD further enhance this contrast between the warmer areas (the sun and clouds) and the cooler areas (the waters), particularly when considering the original painting’s inherent low contrast. DWCA effectively preserves the contrast in the upper warm regions, maintaining a color intensity comparable to that of the original colored version by blending oranges and reds with blues. Conversely, the blue regions lacking significant inherent contrast (e.g., the central right region of the port featuring the crane and water) are primarily preserved only by BT.601 and DWCA.

## 6. Discussion

The primary goal of decolorization is to preserve visual features as much as possible while minimizing information loss and achieving a balanced result where certain aspects are prioritized over others. For example, the CPD model achieves excellent E-score results; however, qualitative evaluation reveals alterations in original color contrast that can affect visual meaning. This is illustrated in Figure 12, for instance, in Image 1, where some methods reduce the gray value of the orange semicircle to a level lower than the background, prioritizing luminance contrast over color contrast. Consequently, the outcome heavily depends on the desired objective, sometimes maintaining contrast even if it introduces distortions in original intensity and, at other times, prioritizing intensity preservation.

In the DWCA results, we observe that *CCPR* values are slightly lower than those of CPD with minimum thresholds above 20; however, qualitative analysis demonstrates a better preservation of color contrasts. At minimum thresholds below 10, there is no significant difference between the two methods. Furthermore, the *CCFR* value for DWCA is notably lower than that of other methods, indicating that DWCA increases contrast in certain regions during grayscale conversion. The critical question then becomes whether this difference represents a meaningful improvement in qualitative assessment. This issue was analyzed in the qualitative evaluation (see Figure 12), where, for example, in Image 1, the semicircle’s intensity increases relative to the background, associating warm colors with lighter tones and cool colors with darker tones —a relationship that is not always present in the original image. In other methods, except NGM, this relationship is not maintained; consequently, the semicircle exhibits a dark-over-light contrast when it is colored, where orange contrasts with violet.

For C2G-SSIM, DWCA achieves values among the highest (above 0.89), with BT.601 being the highest. This suggests that, in addition to color contrast, structural information is also preserved, as confirmed by qualitative analysis.

The Ishihara test highlights the challenges faced by all methods except DWCA when dealing with low-contrast and ambiguous images. Methods employing either global or local contrast analysis or those applying weights or analyzing their channels prove ineffective under these conditions, demonstrating how the warmth–coolness adjustment incorporated in DWCA facilitates improved performance.

Methods performing contrast analysis —both locally and globally (D, NGM, and CPD)— achieve high *CCPR* values; however, qualitative examination of the grayscale conversions reveals an increase in original color contrast that often distorts visual meaning. This is particularly evident in cases with low contrast, such as Monet’s paintings within blue areas, where maintaining the relationship between two hues with a similar value requires increasing their difference during conversion —rendering one darker and the other lighter— without adhering to the warm-light/cool-dark relationship; this can even reverse, as seen in Image 1 (Figure 12). This is particularly ambiguous when perceived by humans, who inherently associate warmth with lightness and coolness with darkness.

Both the relationship between retinal activity and color scale and the adjustment of warmth and coolness represent a novel contribution to the state of the art. This adjustment will enable the development of algorithms that facilitate the automation of parameter configuration, achieving results similar to those obtained in Figure 12 through human calibration. The results demonstrate that DWCA maintains color contrast at levels comparable to other methods and performs better with low contrasts and ambiguous structures, as demonstrated by the Ishihara test. We can conclude the following:

- Quantitatively, DWCA achieves higher *CCPR* and *CCFR* ratios than methods that do not analyze contrast (BT.601 and PCA), slightly surpassing those performing global analysis (D and NGM) and marginally lower compared to those employing local analysis (CPD);
- Qualitatively, the grayscale conversion maintains hue relationships at a high level without distorting the visual meaning of the image;
- As a method that does not perform contrast analysis but relies on the OCC system representing each color and its properties, DWCA yields results independent of local or global circumstances within each image; therefore, the resulting gray value is consistent for each color;
- As a pixel-by-pixel method, it significantly reduces computational cost;
- The warmth/coolness adjustment facilitates grayscale conversion control for both automated processes and human calibration.

## 7. Conclusions and Future Works

This paper presents a novel pixel-by-pixel grayscale conversion method with warmth–coolness adjustment, termed DWCA (Decolorization with Warmth–Coolness Adjustment). This approach is based on the opponent color system (OCC), which combines Schopenhauer’s color theory with principles of retinal anatomy and activity. This system facilitates the definition of a perceptual color scale and allows for intuitive categories such as warm and cool to be managed. The warmth-coolness adjustment in DWCA is based on the dominance of the L channel (red) over the M and S channels (green and blue). This perceptual criterion enables consistent intervention on images, aligning with human perception of color, simplifying both process automation and manual calibration.

DWCA is a low-cost algorithm capable of delivering results comparable —and in many cases superior— to those of more complex state-of-the-art methods. The method demonstrates a particular advantage under conditions of low contrast or chromatic ambiguity, where it preserves relevant information more effectively. In empirical evaluation, the method was subjected to the Ishihara test, widely used for detecting color blindness. DWCA achieved a 100% accuracy rate compared to rates below 50% attained by reference methods.

Finally, in the qualitative analysis, the DWCA method exhibited a notable ability to preserve not only chromatic contrast but also luminance and saturation. Grayscale images produced using this method maintain spatial relationships and patterns between different regions with greater fidelity. This makes DWCA a robust and perceptually coherent solution for grayscale conversion. However, an adjustment of the W and C parameters is necessary to optimize the results, and the creation of algorithms that automate this process is identified as one of the primary avenues for future research.

In conclusion, there is no perfect decolorization method, and all have limitations. Future work aims to reduce these limitations and improve saturation in very light and very dark colors.

## Figures and Tables

**Table 1:** Definition of color categories according to the dominant channels in the ON pathway.

Category	Colors	Dominant Channels
warm	red	L
cool	green and blue	M y S

**Table 2:** *CCPR*, *CCFR*, and E-Score averages in the Color250 dataset for varying minimum contrast thresholds.

	BT.601		PCA		D		NGM		CPD		DWCA	
	**Ave**	σ	**Ave**	σ	**Ave**	σ	**Ave**	σ	**Ave**	σ	**Ave**	σ
τ=4												
*CCPR*	0.86	0.11	0.81	0.13	0.87	0.11	0.84	0.12	0.88	0.10	0.87	0.12
*CCFR*	0.99	0.00	0.99	0.00	0.99	0.01	0.99	0.01	0.99	0.01	0.99	0.01
Escore	0.92	0.07	0.89	0.08	0.92	0.07	0.90	0.08	0.93	0.06	0.92	0.09
τ=10												
*CCPR*	0.76	0.16	0.69	0.17	0.78	0.16	0.75	0.16	0.80	0.15	0.78	0.17
*CCFR*	0.99	0.01	1.00	0.00	0.99	0.01	0.99	0.02	0.99	0.01	0.99	0.03
Escore	0.85	0.11	0.80	0.13	0.86	0.11	0.84	0.11	0.87	0.10	0.85	0.12
τ=20												
*CCPR*	0.64	0.24	0.54	0.23	0.68	0.22	0.65	0.21	0.72	0.20	0.66	0.25
*CCFR*	0.99	0.00	1.00	0.00	0.99	0.04	0.98	0.04	0.99	0.02	0.97	0.05
Escore	0.74	0.20	0.67	0.21	0.78	0.18	0.76	0.17	0.80	0.16	0.74	0.21

**Table 3:** C2G-SSIM averages in the Color250 dataset.

	BT.601		PCA		D		NGM		CPD		DWCA	
	**Ave**	σ	**Ave**	σ	**Ave**	σ	**Ave**	σ	**Ave**	σ	**Ave**	σ
C2G-SSIM	0.96	0.01	0.54	0.43	0.89	0.05	0.74	0.15	0.93	0.04	0.91	0.04

**Table 4:** Accuracy scores on the Ishihara test.

Plates	BT.601	PCA	D	NGM	GPD	DWCA
1 & 38	0/2	0/2	2/2	0/2	2/2	2/2
2–9	6/8	4/8	2/8	2/8	4/8	8/8
10–17	3/8	0/8	4/8	3/8	0/8	8/8
18–21	0/4	0/4	4/4	4/4	0/4	4/4
22–25	0/4	0/4	0/4	1/4	0/4	4/4
26 & 27	0/2	0/2	0/2	0/2	0/2	2/2
28 & 29	2/2	0/2	0/2	0/2	0/2	2/2
30–33	2/4	0/4	2/4	2/4	0/4	4/4
34 & 35	0/2	0/2	0/2	1/2	0/2	2/2
36 & 37	2/2	2/2	1/2	0/2	2/2	2/2
Total	15/38	6/38	17/38	12/38	8/38	38/38
%	39%	15%	44%	31%	21%	100%

## Data Availability

The original contributions presented in this study are included in the article/Supplementary Material. Further inquiries can be directed to the corresponding author(s).

## Supplementary Materials

The following supporting information can be downloaded at: https://www.mdpi.com/article/10.3390/jimaging11060199/s1.
