# Supplementary figures and images for: Decolorization with Warmth–Coolness Adjustment in an Opponent and Complementary Color System (part 2 of 2)
Source: J Imaging. 2025 Jun 18;11(6):199. doi: 10.3390/jimaging11060199 (PMC12194648; doi:10.3390/jimaging11060199)

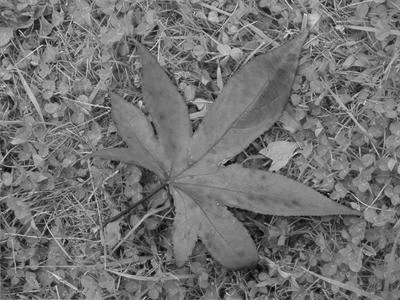

Supplement: Supplementary file 1 [file jimaging-11-00199-s001.zip › DWCA_code/dataset/color250/10_5.png]

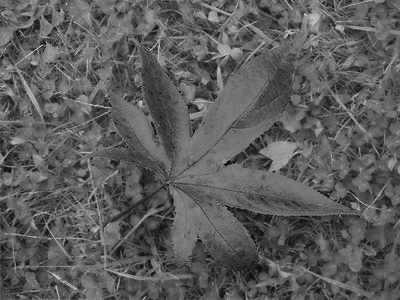

Supplement: Supplementary file 1 [file jimaging-11-00199-s001.zip › DWCA_code/dataset/color250/10_6.png]

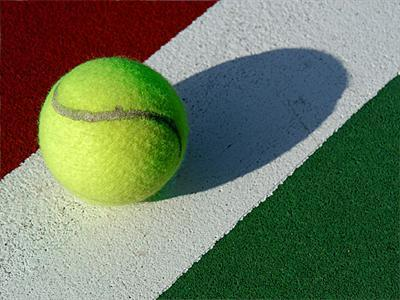

Supplement: Supplementary file 1 [file jimaging-11-00199-s001.zip › DWCA_code/dataset/color250/11.png]

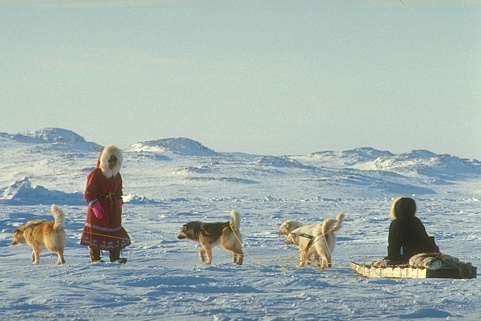

Supplement: Supplementary file 1 [file jimaging-11-00199-s001.zip › DWCA_code/dataset/color250/110.png]

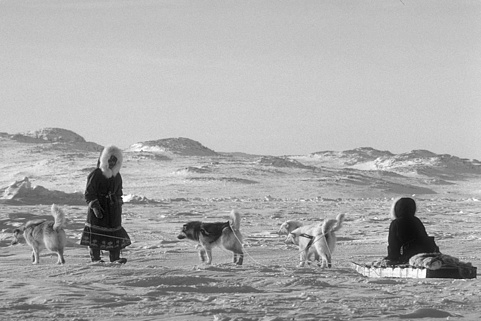

Supplement: Supplementary file 1 [file jimaging-11-00199-s001.zip › DWCA_code/dataset/color250/110_1.png]

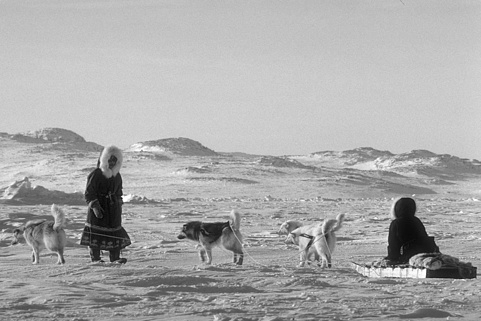

Supplement: Supplementary file 1 [file jimaging-11-00199-s001.zip › DWCA_code/dataset/color250/110_2.png]

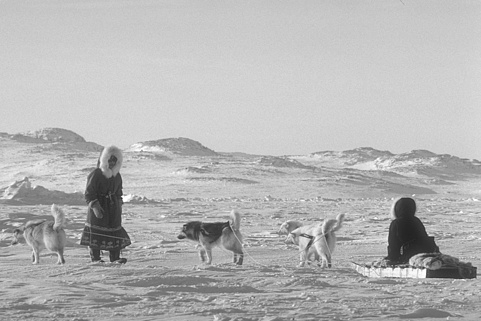

Supplement: Supplementary file 1 [file jimaging-11-00199-s001.zip › DWCA_code/dataset/color250/110_3.png]

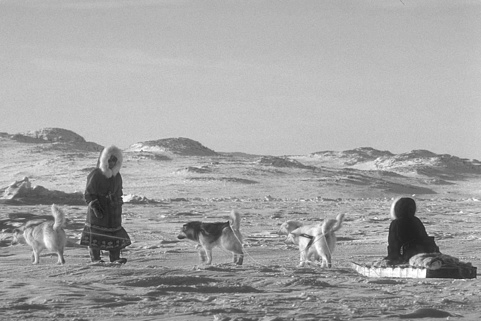

Supplement: Supplementary file 1 [file jimaging-11-00199-s001.zip › DWCA_code/dataset/color250/110_4.png]

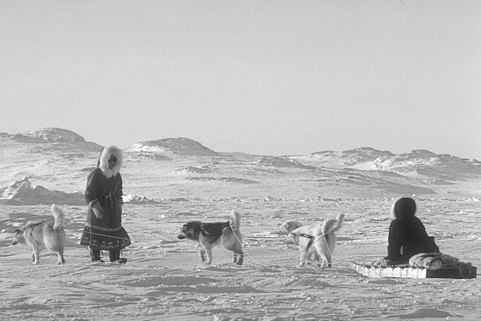

Supplement: Supplementary file 1 [file jimaging-11-00199-s001.zip › DWCA_code/dataset/color250/110_5.png]

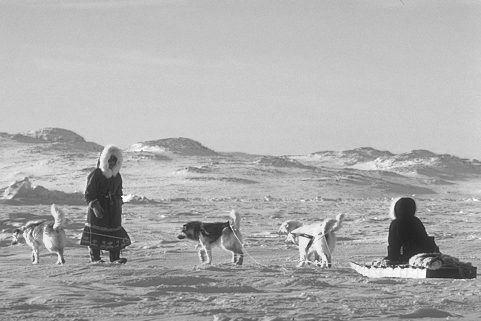

Supplement: Supplementary file 1 [file jimaging-11-00199-s001.zip › DWCA_code/dataset/color250/110_6.png]

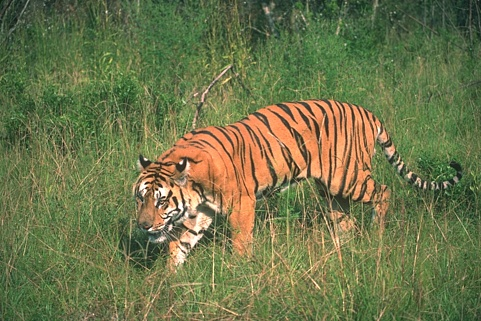

Supplement: Supplementary file 1 [file jimaging-11-00199-s001.zip › DWCA_code/dataset/color250/111.png]

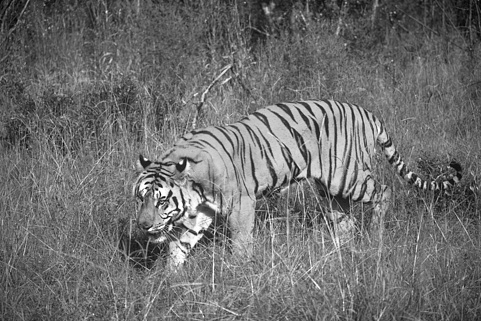

Supplement: Supplementary file 1 [file jimaging-11-00199-s001.zip › DWCA_code/dataset/color250/111_1.png]

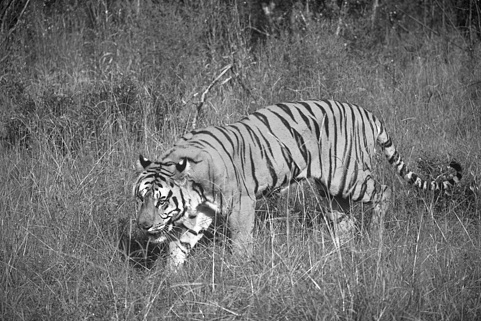

Supplement: Supplementary file 1 [file jimaging-11-00199-s001.zip › DWCA_code/dataset/color250/111_2.png]

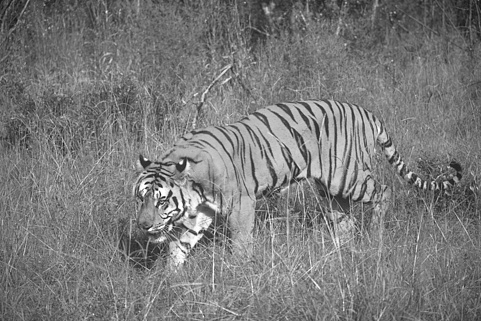

Supplement: Supplementary file 1 [file jimaging-11-00199-s001.zip › DWCA_code/dataset/color250/111_3.png]

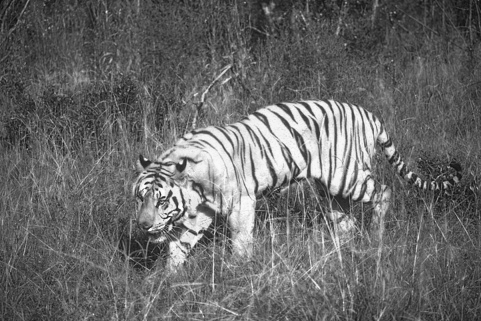

Supplement: Supplementary file 1 [file jimaging-11-00199-s001.zip › DWCA_code/dataset/color250/111_4.png]

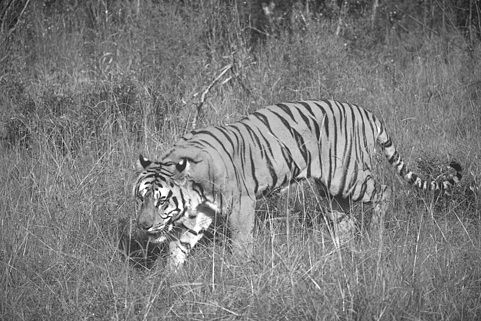

Supplement: Supplementary file 1 [file jimaging-11-00199-s001.zip › DWCA_code/dataset/color250/111_5.png]

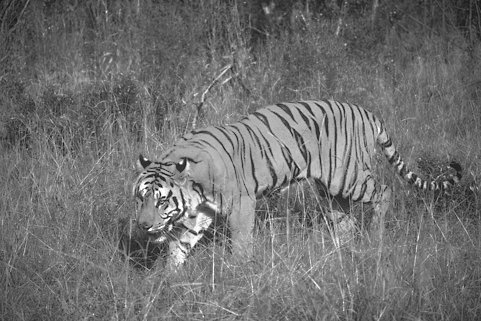

Supplement: Supplementary file 1 [file jimaging-11-00199-s001.zip › DWCA_code/dataset/color250/111_6.png]

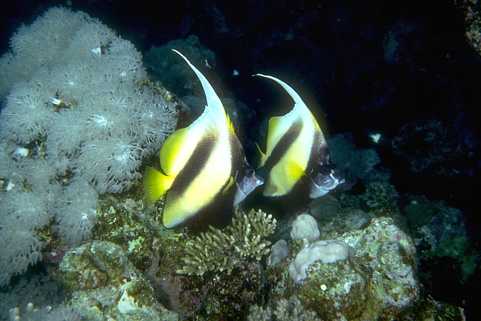

Supplement: Supplementary file 1 [file jimaging-11-00199-s001.zip › DWCA_code/dataset/color250/112.png]

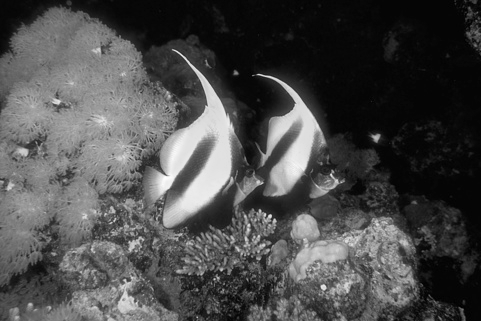

Supplement: Supplementary file 1 [file jimaging-11-00199-s001.zip › DWCA_code/dataset/color250/112_1.png]

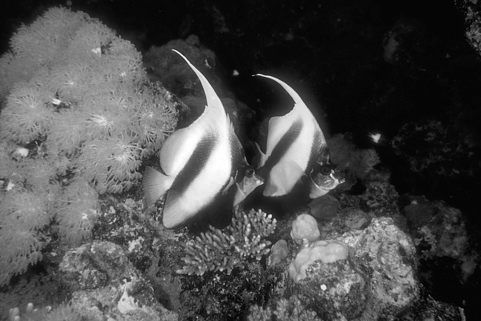

Supplement: Supplementary file 1 [file jimaging-11-00199-s001.zip › DWCA_code/dataset/color250/112_2.png]

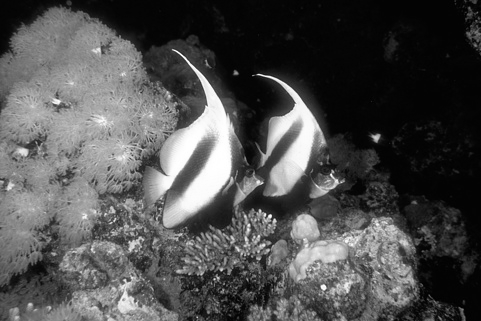

Supplement: Supplementary file 1 [file jimaging-11-00199-s001.zip › DWCA_code/dataset/color250/112_3.png]

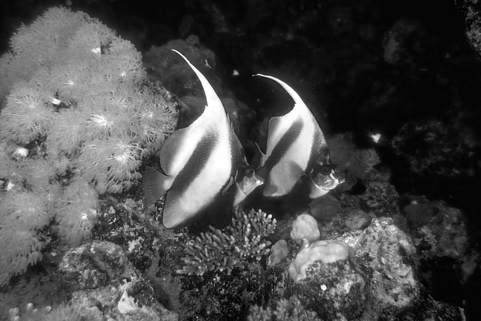

Supplement: Supplementary file 1 [file jimaging-11-00199-s001.zip › DWCA_code/dataset/color250/112_4.png]

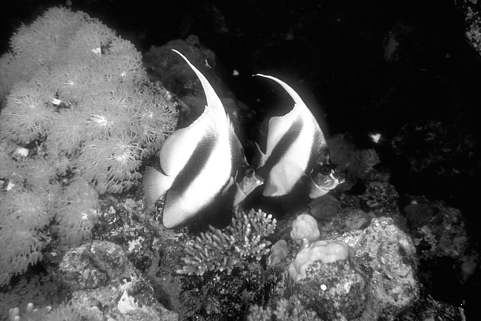

Supplement: Supplementary file 1 [file jimaging-11-00199-s001.zip › DWCA_code/dataset/color250/112_5.png]

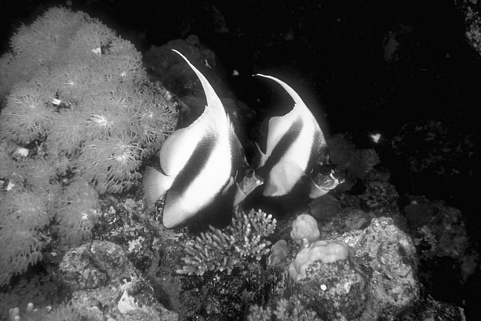

Supplement: Supplementary file 1 [file jimaging-11-00199-s001.zip › DWCA_code/dataset/color250/112_6.png]

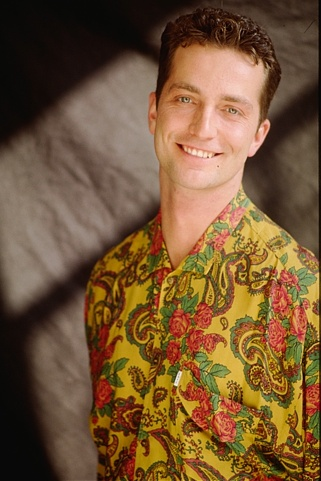

Supplement: Supplementary file 1 [file jimaging-11-00199-s001.zip › DWCA_code/dataset/color250/113.png]

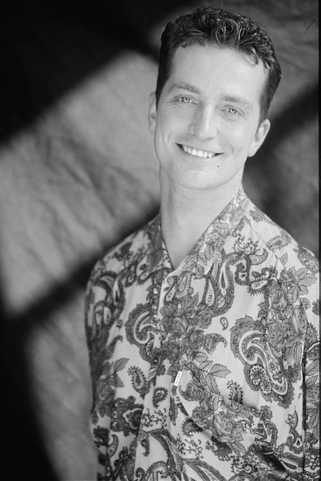

Supplement: Supplementary file 1 [file jimaging-11-00199-s001.zip › DWCA_code/dataset/color250/113_1.png]

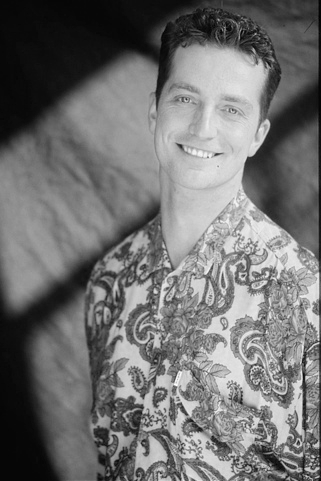

Supplement: Supplementary file 1 [file jimaging-11-00199-s001.zip › DWCA_code/dataset/color250/113_2.png]

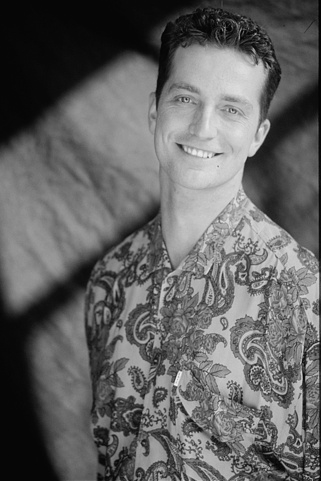

Supplement: Supplementary file 1 [file jimaging-11-00199-s001.zip › DWCA_code/dataset/color250/113_3.png]

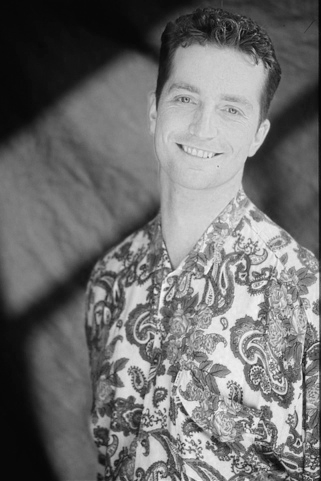

Supplement: Supplementary file 1 [file jimaging-11-00199-s001.zip › DWCA_code/dataset/color250/113_4.png]

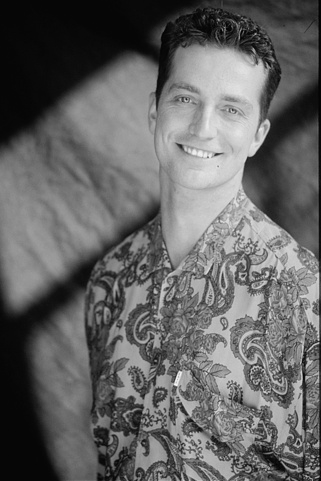

Supplement: Supplementary file 1 [file jimaging-11-00199-s001.zip › DWCA_code/dataset/color250/113_5.png]

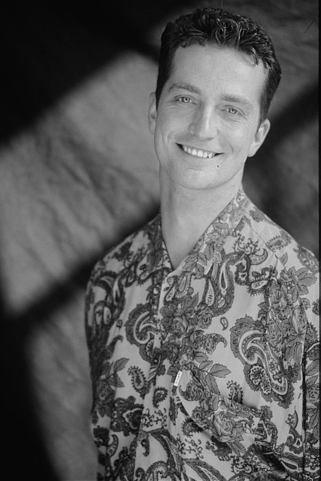

Supplement: Supplementary file 1 [file jimaging-11-00199-s001.zip › DWCA_code/dataset/color250/113_6.png]

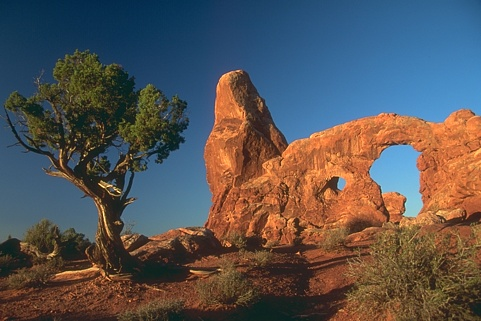

Supplement: Supplementary file 1 [file jimaging-11-00199-s001.zip › DWCA_code/dataset/color250/114.png]

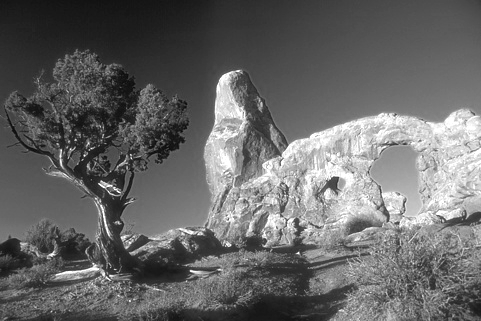

Supplement: Supplementary file 1 [file jimaging-11-00199-s001.zip › DWCA_code/dataset/color250/114_1.png]

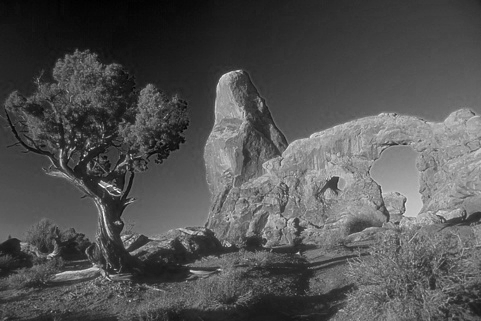

Supplement: Supplementary file 1 [file jimaging-11-00199-s001.zip › DWCA_code/dataset/color250/114_2.png]

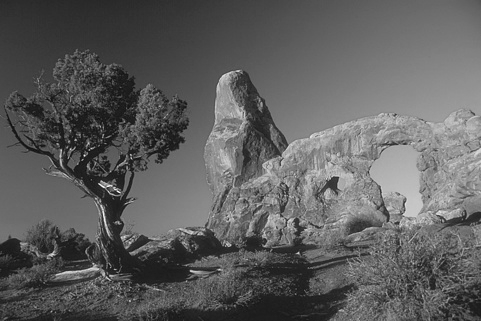

Supplement: Supplementary file 1 [file jimaging-11-00199-s001.zip › DWCA_code/dataset/color250/114_3.png]

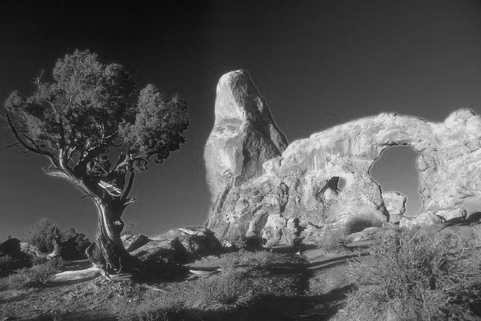

Supplement: Supplementary file 1 [file jimaging-11-00199-s001.zip › DWCA_code/dataset/color250/114_4.png]

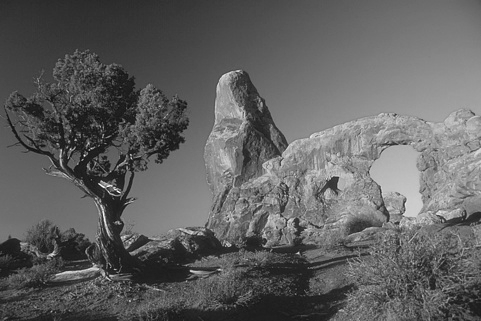

Supplement: Supplementary file 1 [file jimaging-11-00199-s001.zip › DWCA_code/dataset/color250/114_5.png]

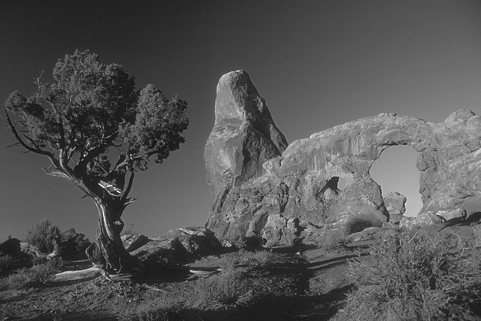

Supplement: Supplementary file 1 [file jimaging-11-00199-s001.zip › DWCA_code/dataset/color250/114_6.png]

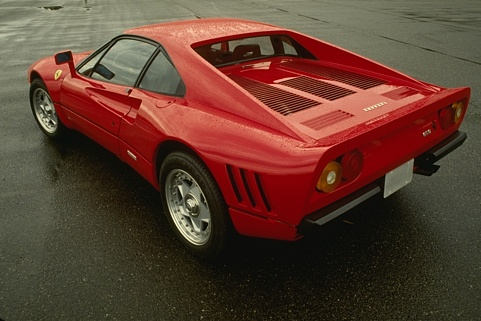

Supplement: Supplementary file 1 [file jimaging-11-00199-s001.zip › DWCA_code/dataset/color250/115.png]

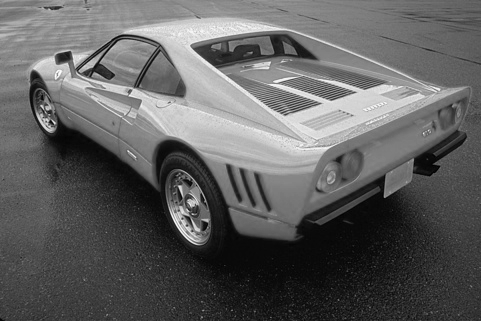

Supplement: Supplementary file 1 [file jimaging-11-00199-s001.zip › DWCA_code/dataset/color250/115_1.png]

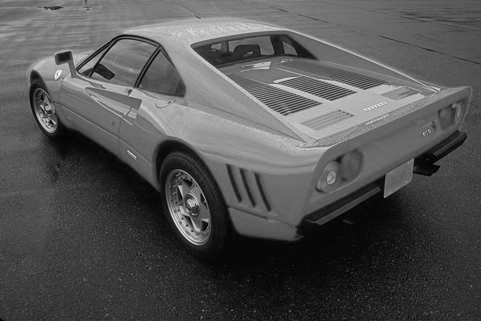

Supplement: Supplementary file 1 [file jimaging-11-00199-s001.zip › DWCA_code/dataset/color250/115_2.png]

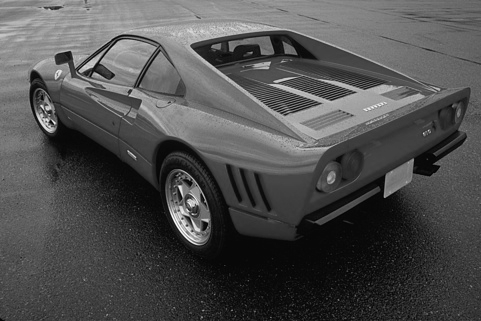

Supplement: Supplementary file 1 [file jimaging-11-00199-s001.zip › DWCA_code/dataset/color250/115_3.png]

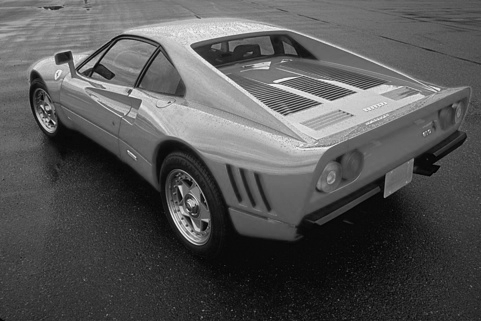

Supplement: Supplementary file 1 [file jimaging-11-00199-s001.zip › DWCA_code/dataset/color250/115_4.png]

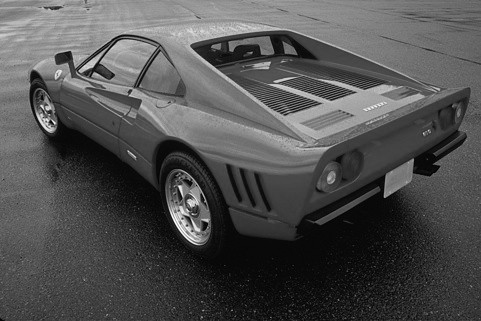

Supplement: Supplementary file 1 [file jimaging-11-00199-s001.zip › DWCA_code/dataset/color250/115_5.png]

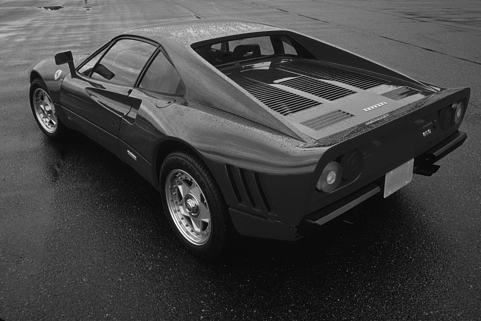

Supplement: Supplementary file 1 [file jimaging-11-00199-s001.zip › DWCA_code/dataset/color250/115_6.png]

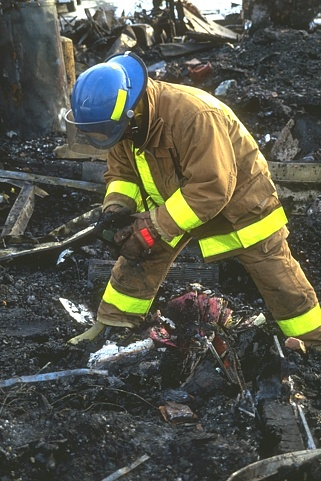

Supplement: Supplementary file 1 [file jimaging-11-00199-s001.zip › DWCA_code/dataset/color250/116.png]

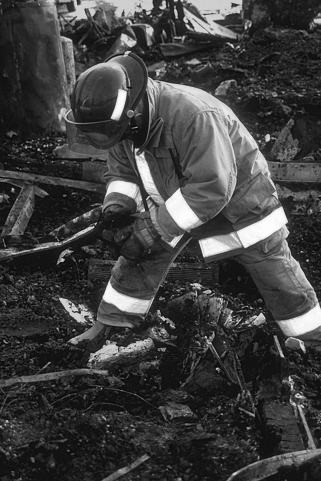

Supplement: Supplementary file 1 [file jimaging-11-00199-s001.zip › DWCA_code/dataset/color250/116_1.png]

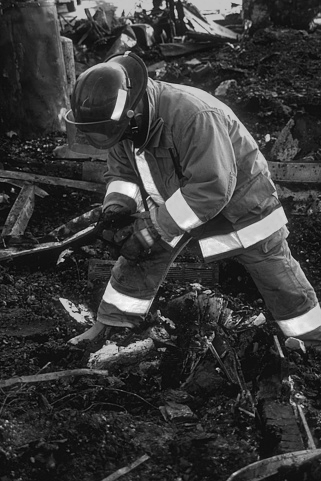

Supplement: Supplementary file 1 [file jimaging-11-00199-s001.zip › DWCA_code/dataset/color250/116_2.png]

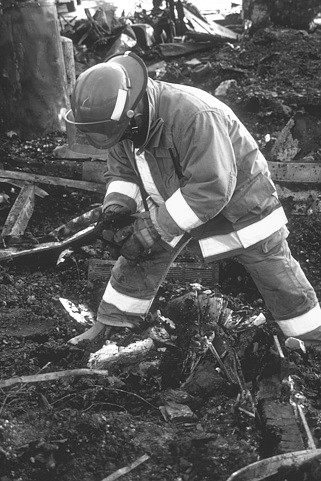

Supplement: Supplementary file 1 [file jimaging-11-00199-s001.zip › DWCA_code/dataset/color250/116_3.png]

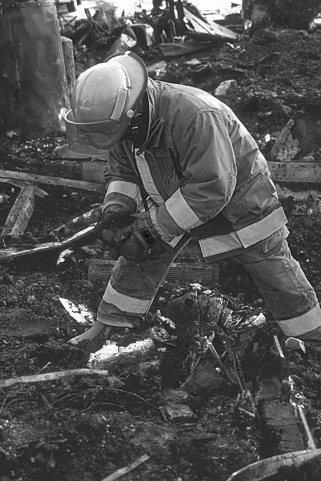

Supplement: Supplementary file 1 [file jimaging-11-00199-s001.zip › DWCA_code/dataset/color250/116_4.png]

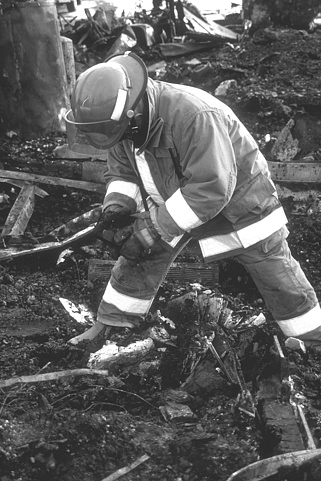

Supplement: Supplementary file 1 [file jimaging-11-00199-s001.zip › DWCA_code/dataset/color250/116_5.png]

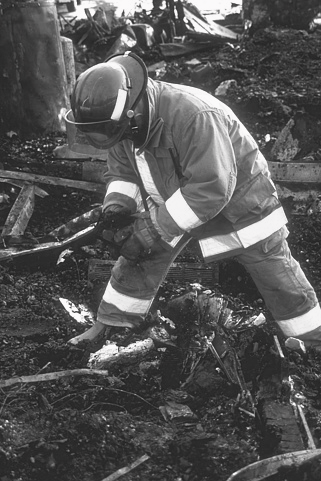

Supplement: Supplementary file 1 [file jimaging-11-00199-s001.zip › DWCA_code/dataset/color250/116_6.png]

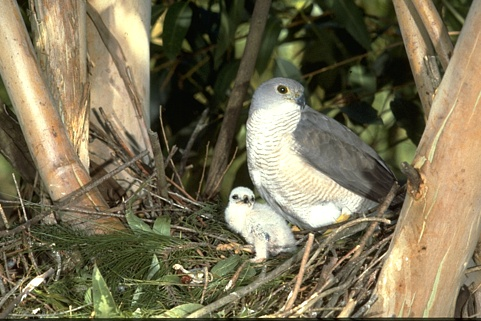

Supplement: Supplementary file 1 [file jimaging-11-00199-s001.zip › DWCA_code/dataset/color250/117.png]

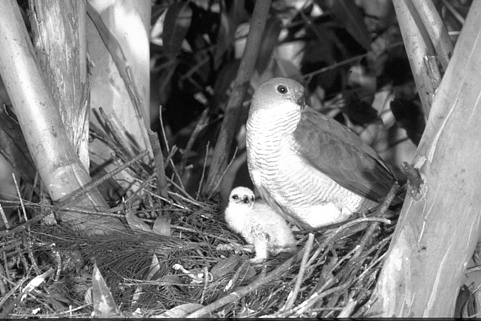

Supplement: Supplementary file 1 [file jimaging-11-00199-s001.zip › DWCA_code/dataset/color250/117_1.png]

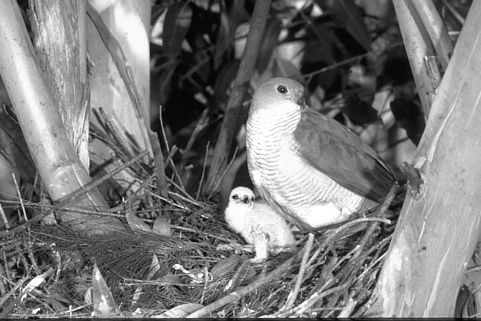

Supplement: Supplementary file 1 [file jimaging-11-00199-s001.zip › DWCA_code/dataset/color250/117_2.png]

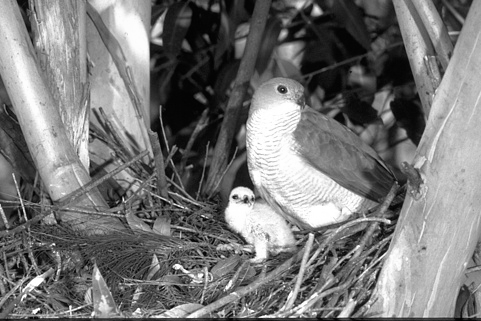

Supplement: Supplementary file 1 [file jimaging-11-00199-s001.zip › DWCA_code/dataset/color250/117_3.png]

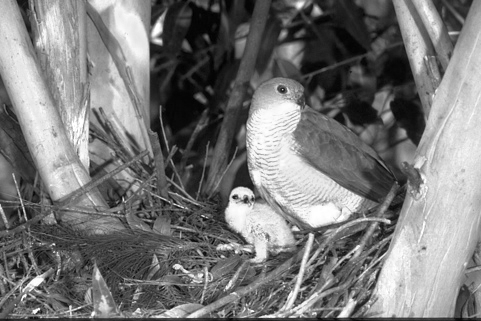

Supplement: Supplementary file 1 [file jimaging-11-00199-s001.zip › DWCA_code/dataset/color250/117_4.png]

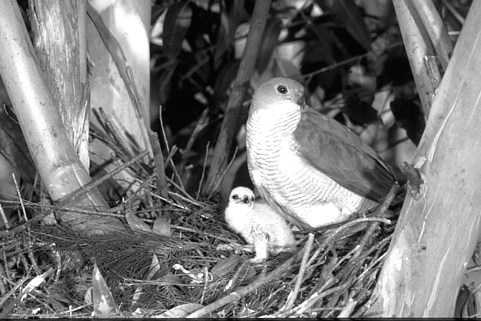

Supplement: Supplementary file 1 [file jimaging-11-00199-s001.zip › DWCA_code/dataset/color250/117_5.png]

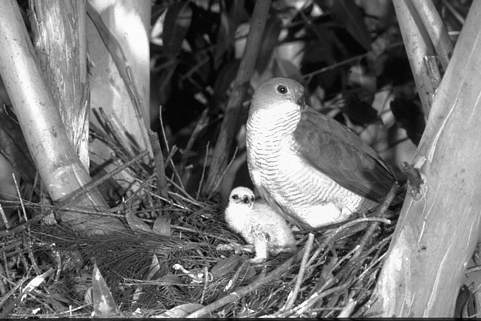

Supplement: Supplementary file 1 [file jimaging-11-00199-s001.zip › DWCA_code/dataset/color250/117_6.png]

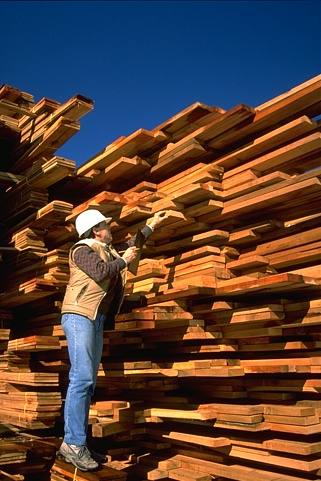

Supplement: Supplementary file 1 [file jimaging-11-00199-s001.zip › DWCA_code/dataset/color250/118.png]

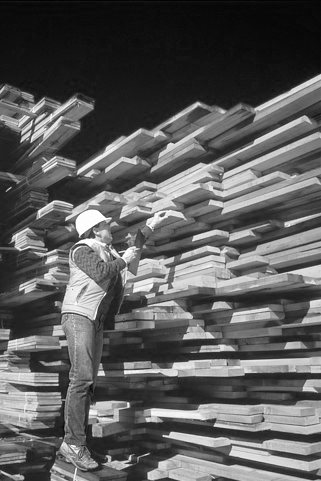

Supplement: Supplementary file 1 [file jimaging-11-00199-s001.zip › DWCA_code/dataset/color250/118_1.png]

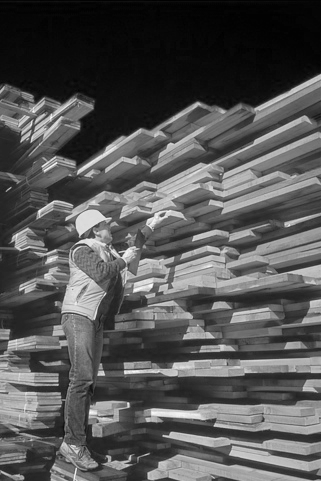

Supplement: Supplementary file 1 [file jimaging-11-00199-s001.zip › DWCA_code/dataset/color250/118_2.png]

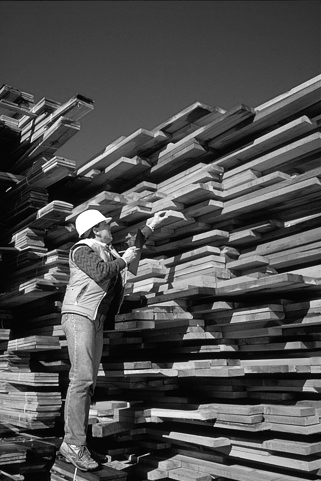

Supplement: Supplementary file 1 [file jimaging-11-00199-s001.zip › DWCA_code/dataset/color250/118_3.png]

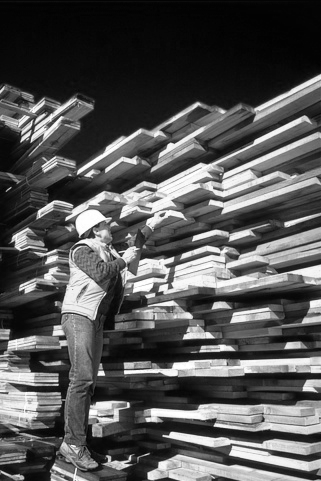

Supplement: Supplementary file 1 [file jimaging-11-00199-s001.zip › DWCA_code/dataset/color250/118_4.png]

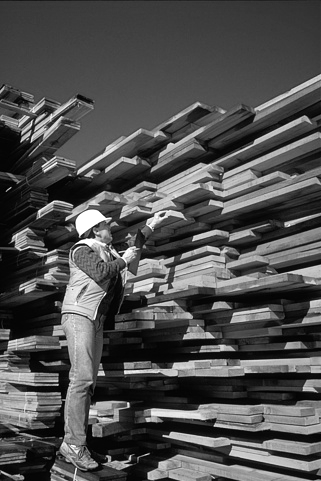

Supplement: Supplementary file 1 [file jimaging-11-00199-s001.zip › DWCA_code/dataset/color250/118_5.png]

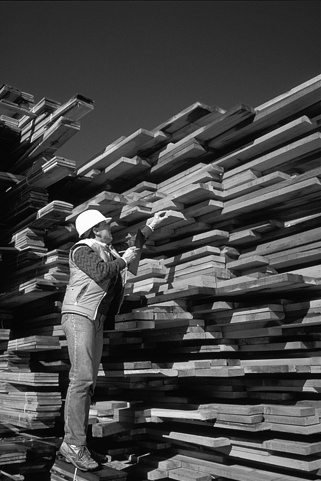

Supplement: Supplementary file 1 [file jimaging-11-00199-s001.zip › DWCA_code/dataset/color250/118_6.png]

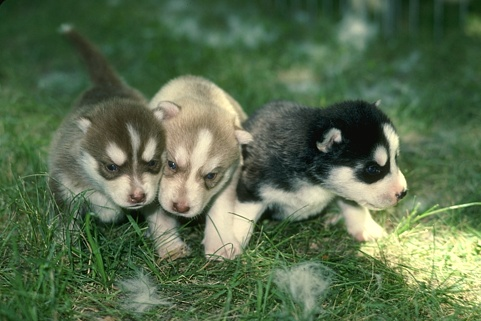

Supplement: Supplementary file 1 [file jimaging-11-00199-s001.zip › DWCA_code/dataset/color250/119.png]

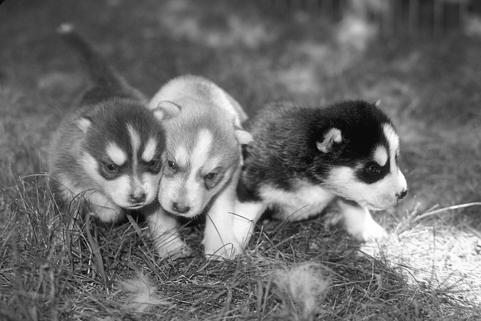

Supplement: Supplementary file 1 [file jimaging-11-00199-s001.zip › DWCA_code/dataset/color250/119_1.png]

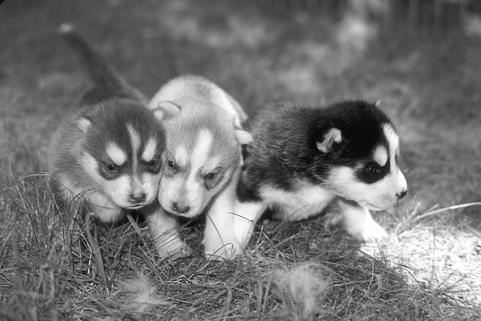

Supplement: Supplementary file 1 [file jimaging-11-00199-s001.zip › DWCA_code/dataset/color250/119_2.png]

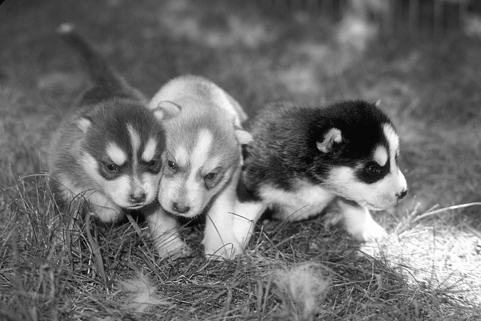

Supplement: Supplementary file 1 [file jimaging-11-00199-s001.zip › DWCA_code/dataset/color250/119_3.png]

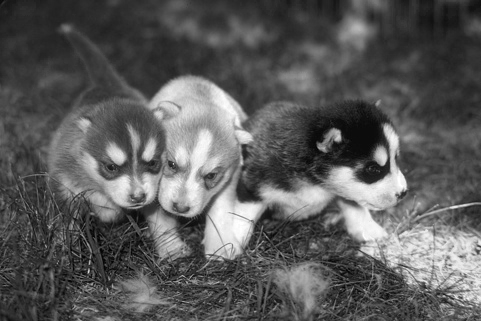

Supplement: Supplementary file 1 [file jimaging-11-00199-s001.zip › DWCA_code/dataset/color250/119_4.png]

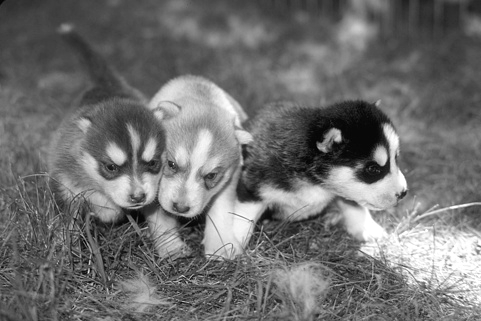

Supplement: Supplementary file 1 [file jimaging-11-00199-s001.zip › DWCA_code/dataset/color250/119_5.png]

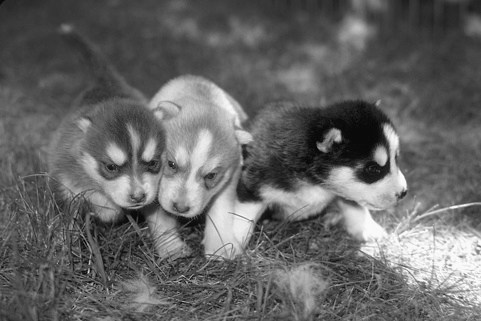

Supplement: Supplementary file 1 [file jimaging-11-00199-s001.zip › DWCA_code/dataset/color250/119_6.png]

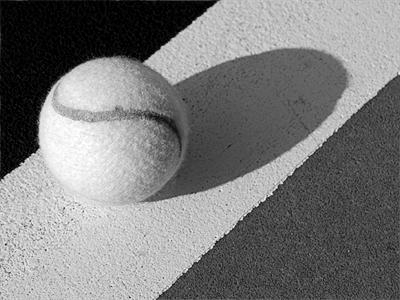

Supplement: Supplementary file 1 [file jimaging-11-00199-s001.zip › DWCA_code/dataset/color250/11_1.png]

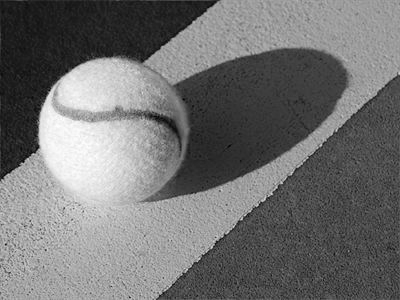

Supplement: Supplementary file 1 [file jimaging-11-00199-s001.zip › DWCA_code/dataset/color250/11_2.png]

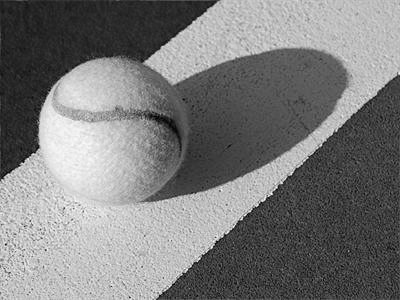

Supplement: Supplementary file 1 [file jimaging-11-00199-s001.zip › DWCA_code/dataset/color250/11_3.png]

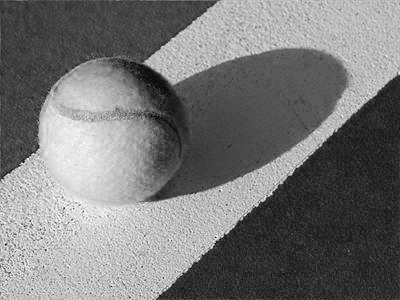

Supplement: Supplementary file 1 [file jimaging-11-00199-s001.zip › DWCA_code/dataset/color250/11_4.png]

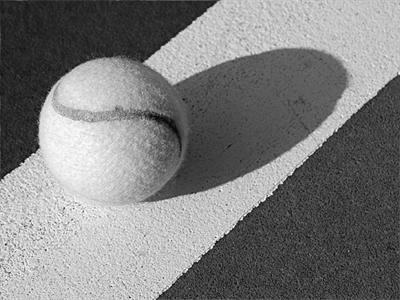

Supplement: Supplementary file 1 [file jimaging-11-00199-s001.zip › DWCA_code/dataset/color250/11_5.png]

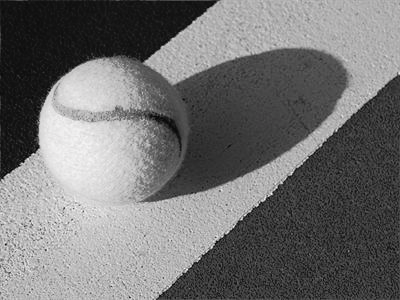

Supplement: Supplementary file 1 [file jimaging-11-00199-s001.zip › DWCA_code/dataset/color250/11_6.png]

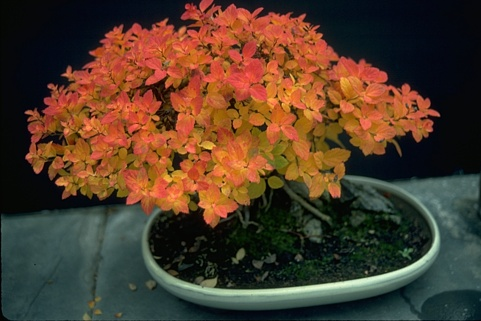

Supplement: Supplementary file 1 [file jimaging-11-00199-s001.zip › DWCA_code/dataset/color250/12.png]

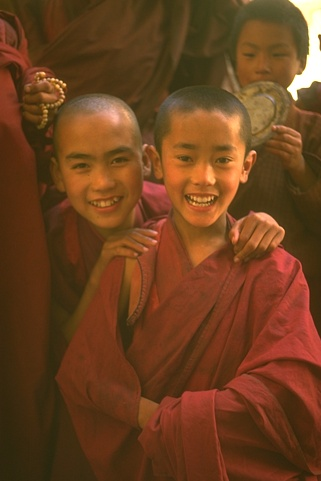

Supplement: Supplementary file 1 [file jimaging-11-00199-s001.zip › DWCA_code/dataset/color250/120.png]

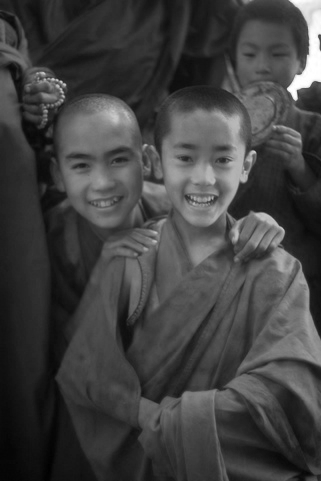

Supplement: Supplementary file 1 [file jimaging-11-00199-s001.zip › DWCA_code/dataset/color250/120_1.png]

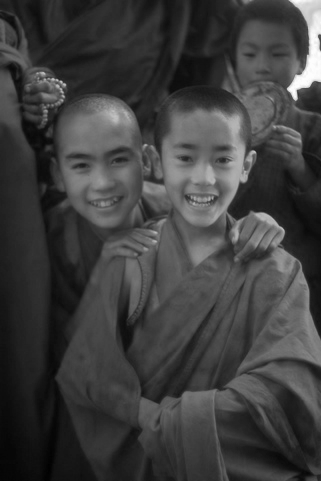

Supplement: Supplementary file 1 [file jimaging-11-00199-s001.zip › DWCA_code/dataset/color250/120_2.png]

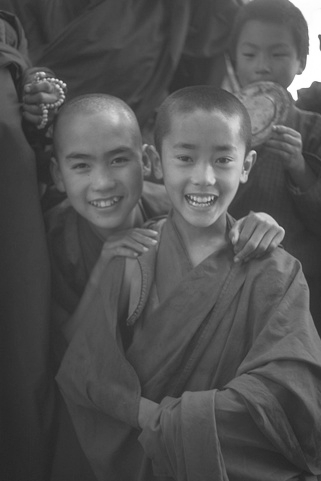

Supplement: Supplementary file 1 [file jimaging-11-00199-s001.zip › DWCA_code/dataset/color250/120_3.png]

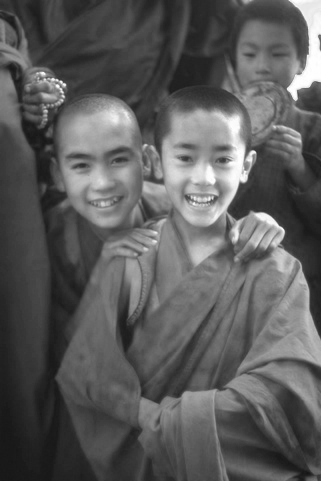

Supplement: Supplementary file 1 [file jimaging-11-00199-s001.zip › DWCA_code/dataset/color250/120_4.png]

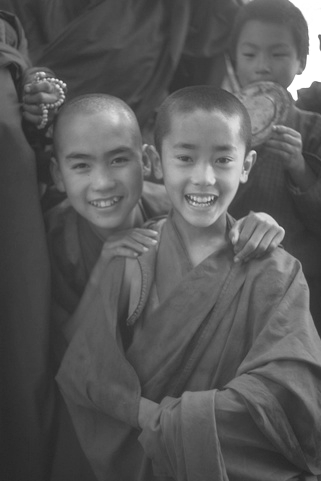

Supplement: Supplementary file 1 [file jimaging-11-00199-s001.zip › DWCA_code/dataset/color250/120_5.png]

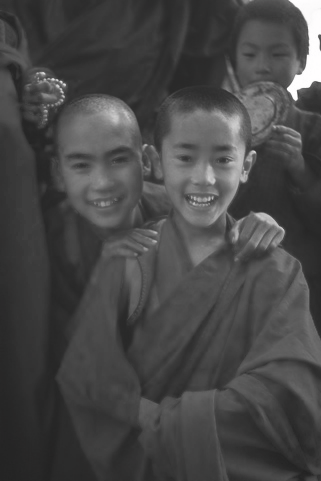

Supplement: Supplementary file 1 [file jimaging-11-00199-s001.zip › DWCA_code/dataset/color250/120_6.png]

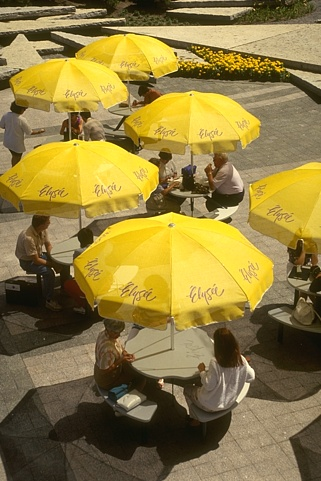

Supplement: Supplementary file 1 [file jimaging-11-00199-s001.zip › DWCA_code/dataset/color250/121.png]

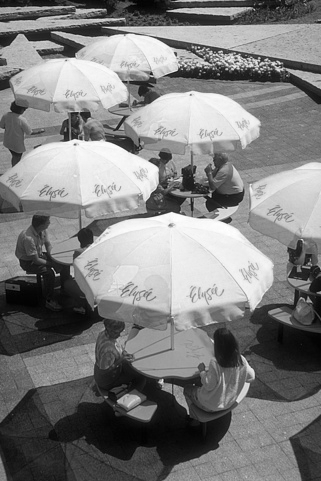

Supplement: Supplementary file 1 [file jimaging-11-00199-s001.zip › DWCA_code/dataset/color250/121_1.png]

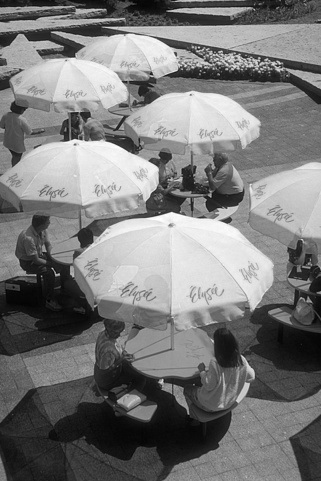

Supplement: Supplementary file 1 [file jimaging-11-00199-s001.zip › DWCA_code/dataset/color250/121_2.png]

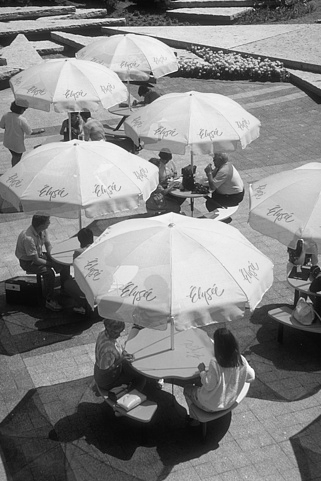

Supplement: Supplementary file 1 [file jimaging-11-00199-s001.zip › DWCA_code/dataset/color250/121_3.png]

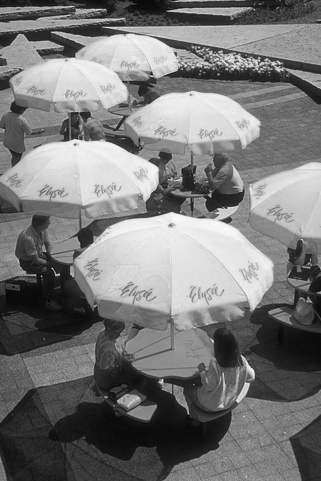

Supplement: Supplementary file 1 [file jimaging-11-00199-s001.zip › DWCA_code/dataset/color250/121_4.png]

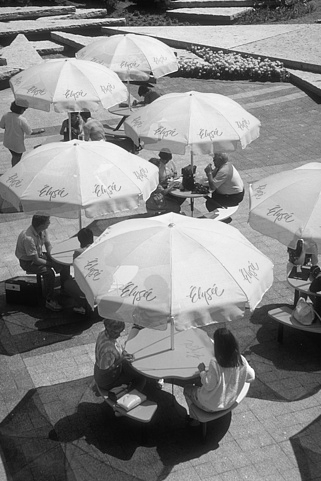

Supplement: Supplementary file 1 [file jimaging-11-00199-s001.zip › DWCA_code/dataset/color250/121_5.png]

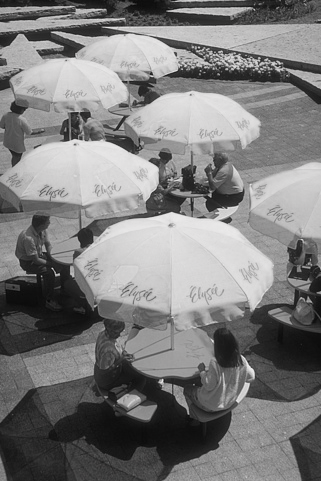

Supplement: Supplementary file 1 [file jimaging-11-00199-s001.zip › DWCA_code/dataset/color250/121_6.png]

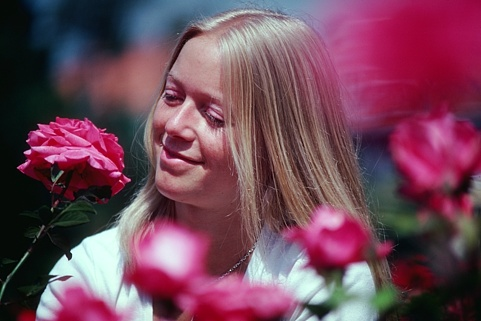

Supplement: Supplementary file 1 [file jimaging-11-00199-s001.zip › DWCA_code/dataset/color250/122.png]

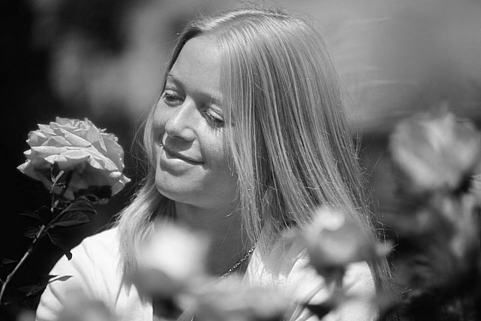

Supplement: Supplementary file 1 [file jimaging-11-00199-s001.zip › DWCA_code/dataset/color250/122_1.png]

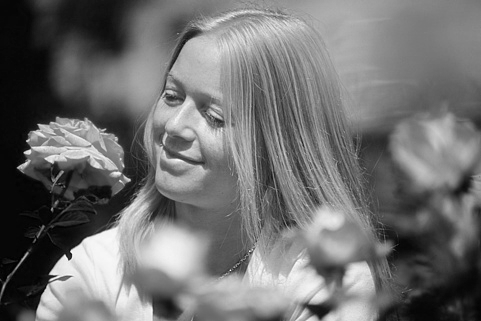

Supplement: Supplementary file 1 [file jimaging-11-00199-s001.zip › DWCA_code/dataset/color250/122_2.png]

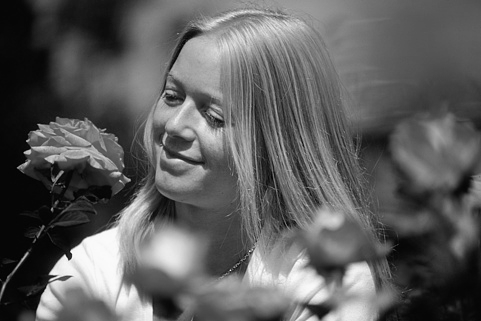

Supplement: Supplementary file 1 [file jimaging-11-00199-s001.zip › DWCA_code/dataset/color250/122_3.png]

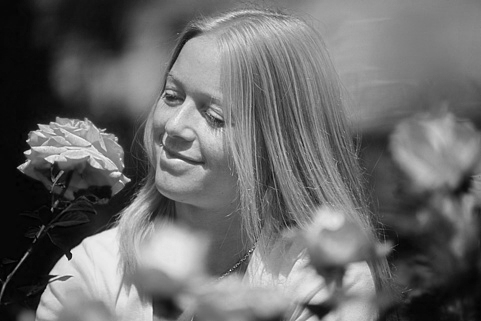

Supplement: Supplementary file 1 [file jimaging-11-00199-s001.zip › DWCA_code/dataset/color250/122_4.png]

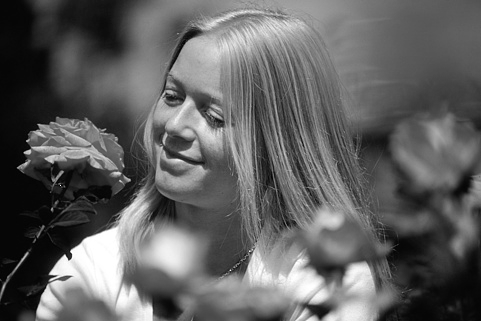

Supplement: Supplementary file 1 [file jimaging-11-00199-s001.zip › DWCA_code/dataset/color250/122_5.png]
