# Supplementary figures and images for: Decolorization with Warmth–Coolness Adjustment in an Opponent and Complementary Color System (part 1 of 2)
Source: J Imaging. 2025 Jun 18;11(6):199. doi: 10.3390/jimaging11060199 (PMC12194648; doi:10.3390/jimaging11060199)

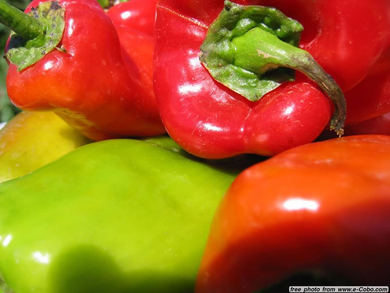

Supplement: Supplementary file 1 [file jimaging-11-00199-s001.zip › DWCA_code/dataset/cadik/original/1.png]

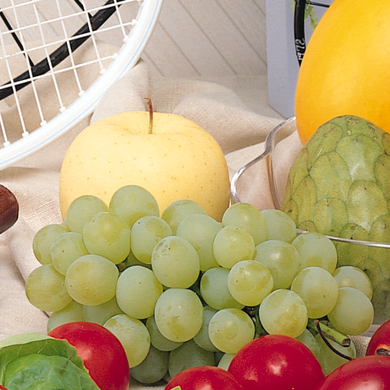

Supplement: Supplementary file 1 [file jimaging-11-00199-s001.zip › DWCA_code/dataset/cadik/original/10.png]

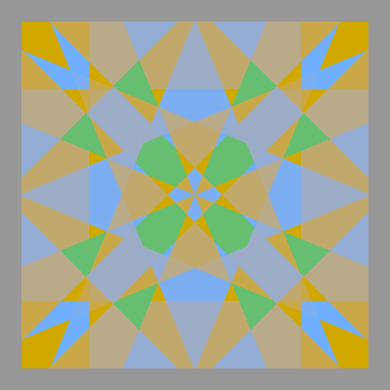

Supplement: Supplementary file 1 [file jimaging-11-00199-s001.zip › DWCA_code/dataset/cadik/original/11.png]

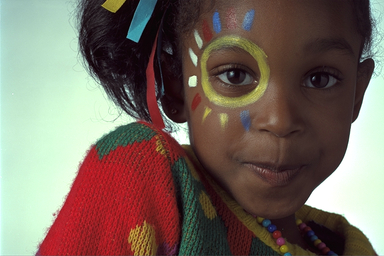

Supplement: Supplementary file 1 [file jimaging-11-00199-s001.zip › DWCA_code/dataset/cadik/original/12.png]

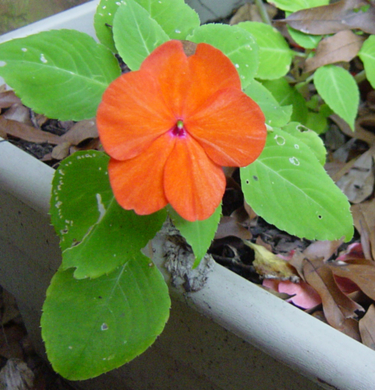

Supplement: Supplementary file 1 [file jimaging-11-00199-s001.zip › DWCA_code/dataset/cadik/original/13.png]

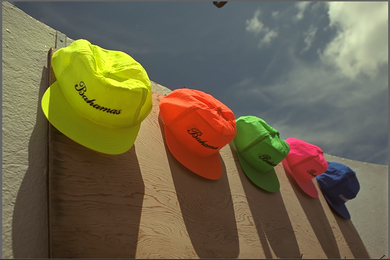

Supplement: Supplementary file 1 [file jimaging-11-00199-s001.zip › DWCA_code/dataset/cadik/original/14.png]

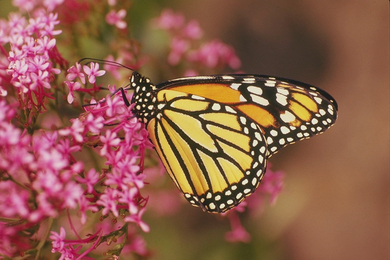

Supplement: Supplementary file 1 [file jimaging-11-00199-s001.zip › DWCA_code/dataset/cadik/original/15.png]

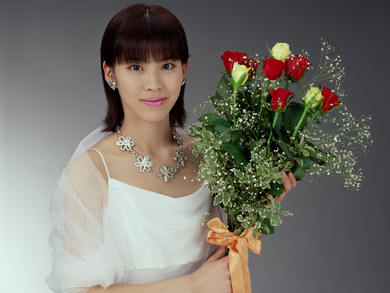

Supplement: Supplementary file 1 [file jimaging-11-00199-s001.zip › DWCA_code/dataset/cadik/original/16.png]

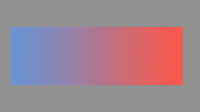

Supplement: Supplementary file 1 [file jimaging-11-00199-s001.zip › DWCA_code/dataset/cadik/original/17.png]

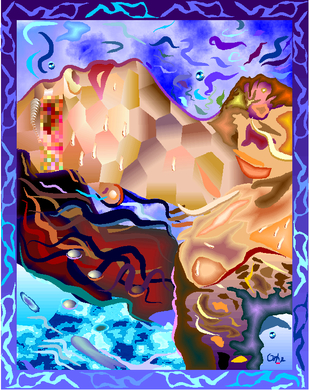

Supplement: Supplementary file 1 [file jimaging-11-00199-s001.zip › DWCA_code/dataset/cadik/original/18.png]

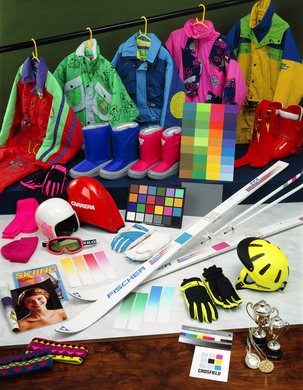

Supplement: Supplementary file 1 [file jimaging-11-00199-s001.zip › DWCA_code/dataset/cadik/original/19.png]

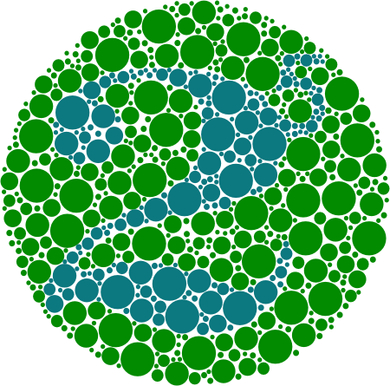

Supplement: Supplementary file 1 [file jimaging-11-00199-s001.zip › DWCA_code/dataset/cadik/original/2.png]

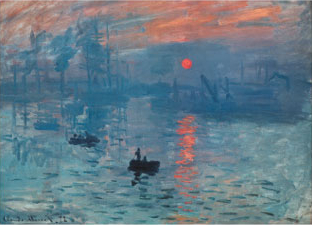

Supplement: Supplementary file 1 [file jimaging-11-00199-s001.zip › DWCA_code/dataset/cadik/original/20.png]

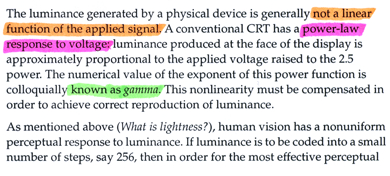

Supplement: Supplementary file 1 [file jimaging-11-00199-s001.zip › DWCA_code/dataset/cadik/original/21.png]

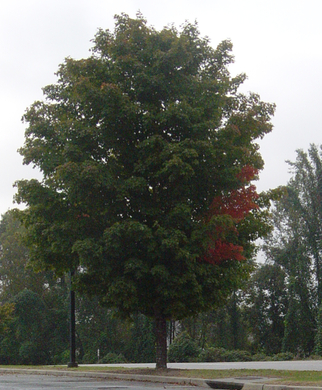

Supplement: Supplementary file 1 [file jimaging-11-00199-s001.zip › DWCA_code/dataset/cadik/original/22.png]

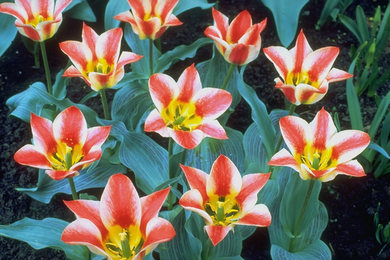

Supplement: Supplementary file 1 [file jimaging-11-00199-s001.zip › DWCA_code/dataset/cadik/original/23.png]

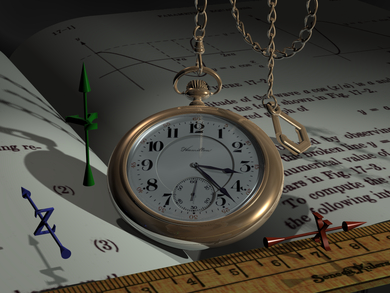

Supplement: Supplementary file 1 [file jimaging-11-00199-s001.zip › DWCA_code/dataset/cadik/original/24.png]

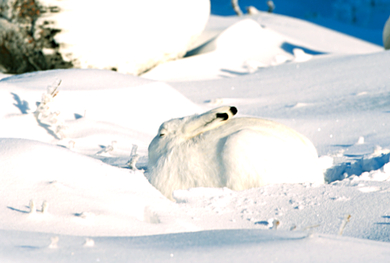

Supplement: Supplementary file 1 [file jimaging-11-00199-s001.zip › DWCA_code/dataset/cadik/original/3.png]

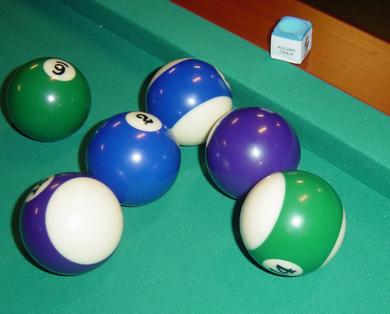

Supplement: Supplementary file 1 [file jimaging-11-00199-s001.zip › DWCA_code/dataset/cadik/original/4.png]

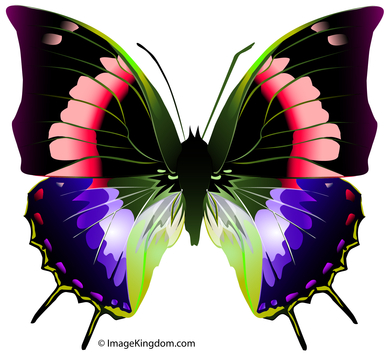

Supplement: Supplementary file 1 [file jimaging-11-00199-s001.zip › DWCA_code/dataset/cadik/original/5.png]

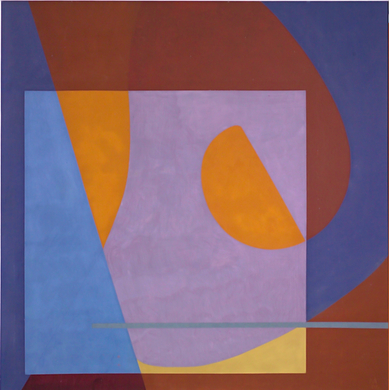

Supplement: Supplementary file 1 [file jimaging-11-00199-s001.zip › DWCA_code/dataset/cadik/original/6.png]

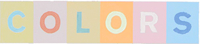

Supplement: Supplementary file 1 [file jimaging-11-00199-s001.zip › DWCA_code/dataset/cadik/original/7.png]

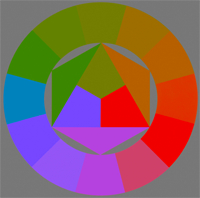

Supplement: Supplementary file 1 [file jimaging-11-00199-s001.zip › DWCA_code/dataset/cadik/original/8.png]

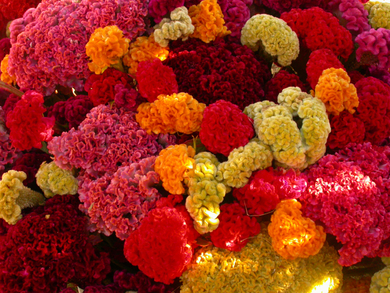

Supplement: Supplementary file 1 [file jimaging-11-00199-s001.zip › DWCA_code/dataset/cadik/original/9.png]

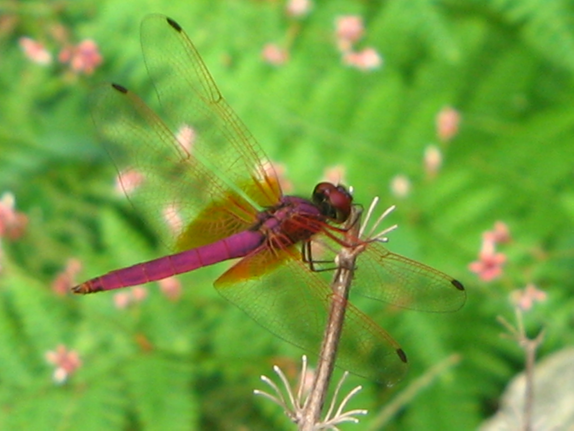

Supplement: Supplementary file 1 [file jimaging-11-00199-s001.zip › DWCA_code/dataset/color250/1.png]

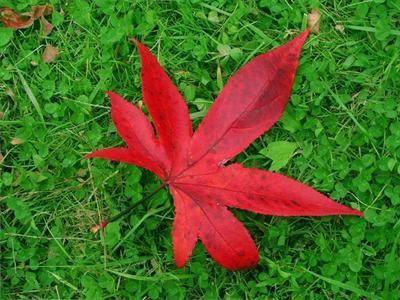

Supplement: Supplementary file 1 [file jimaging-11-00199-s001.zip › DWCA_code/dataset/color250/10.png]

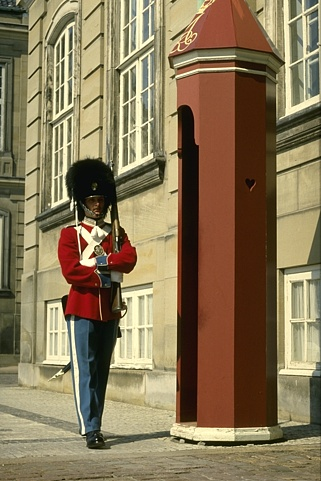

Supplement: Supplementary file 1 [file jimaging-11-00199-s001.zip › DWCA_code/dataset/color250/100.png]

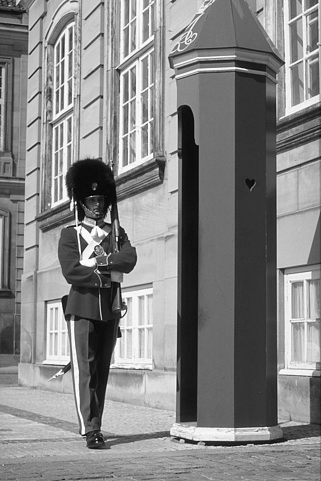

Supplement: Supplementary file 1 [file jimaging-11-00199-s001.zip › DWCA_code/dataset/color250/100_1.png]

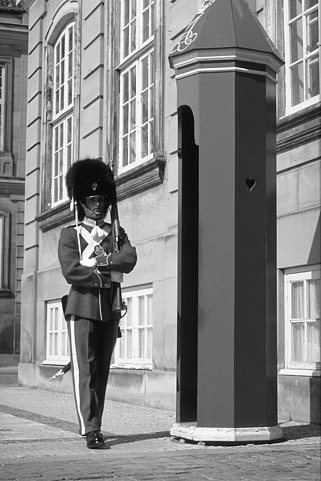

Supplement: Supplementary file 1 [file jimaging-11-00199-s001.zip › DWCA_code/dataset/color250/100_2.png]

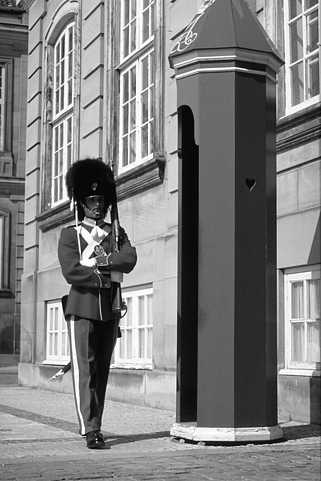

Supplement: Supplementary file 1 [file jimaging-11-00199-s001.zip › DWCA_code/dataset/color250/100_3.png]

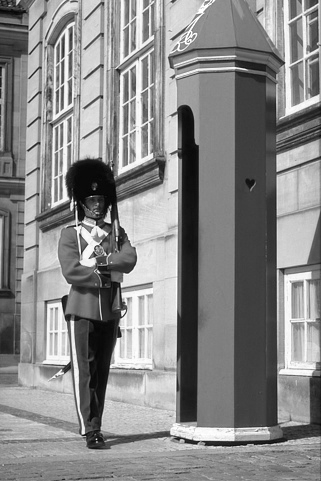

Supplement: Supplementary file 1 [file jimaging-11-00199-s001.zip › DWCA_code/dataset/color250/100_4.png]

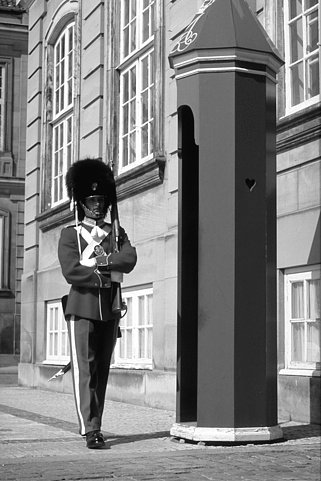

Supplement: Supplementary file 1 [file jimaging-11-00199-s001.zip › DWCA_code/dataset/color250/100_5.png]

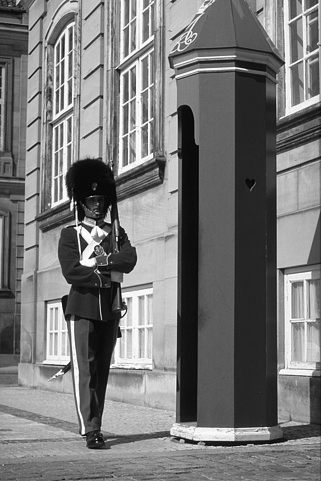

Supplement: Supplementary file 1 [file jimaging-11-00199-s001.zip › DWCA_code/dataset/color250/100_6.png]

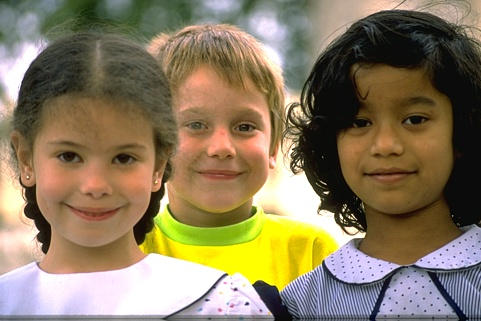

Supplement: Supplementary file 1 [file jimaging-11-00199-s001.zip › DWCA_code/dataset/color250/101.png]

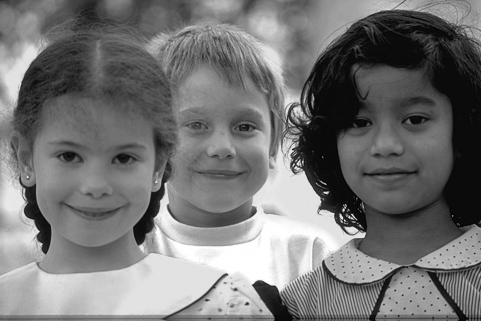

Supplement: Supplementary file 1 [file jimaging-11-00199-s001.zip › DWCA_code/dataset/color250/101_1.png]

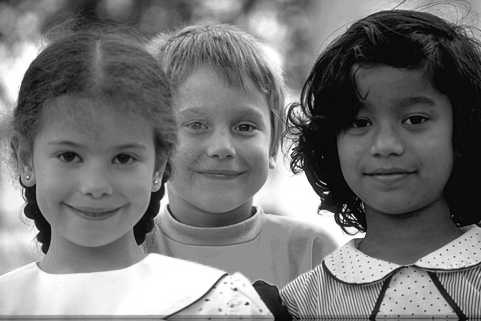

Supplement: Supplementary file 1 [file jimaging-11-00199-s001.zip › DWCA_code/dataset/color250/101_2.png]

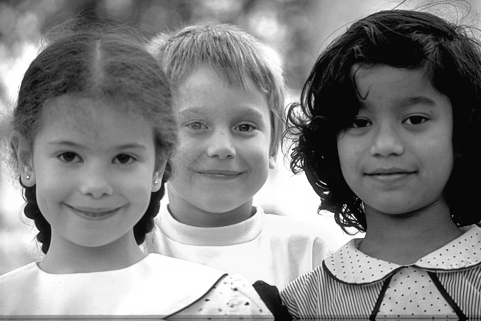

Supplement: Supplementary file 1 [file jimaging-11-00199-s001.zip › DWCA_code/dataset/color250/101_3.png]

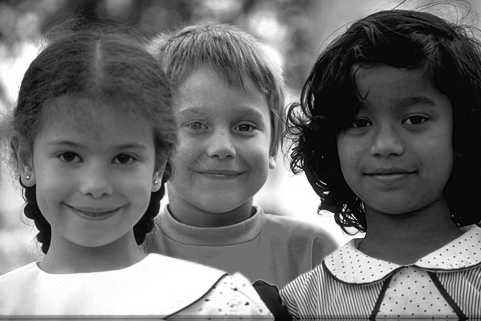

Supplement: Supplementary file 1 [file jimaging-11-00199-s001.zip › DWCA_code/dataset/color250/101_4.png]

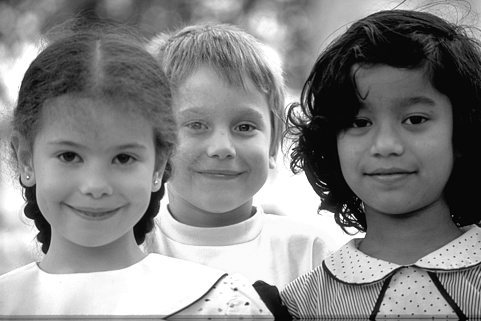

Supplement: Supplementary file 1 [file jimaging-11-00199-s001.zip › DWCA_code/dataset/color250/101_5.png]

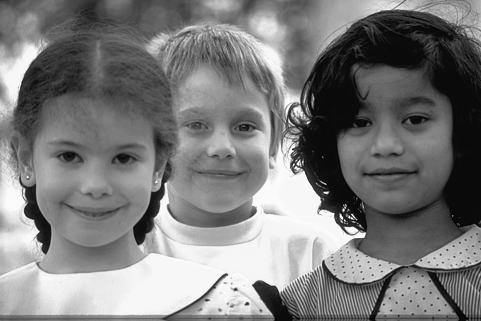

Supplement: Supplementary file 1 [file jimaging-11-00199-s001.zip › DWCA_code/dataset/color250/101_6.png]

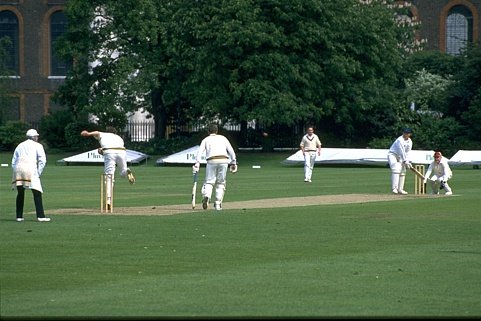

Supplement: Supplementary file 1 [file jimaging-11-00199-s001.zip › DWCA_code/dataset/color250/102.png]

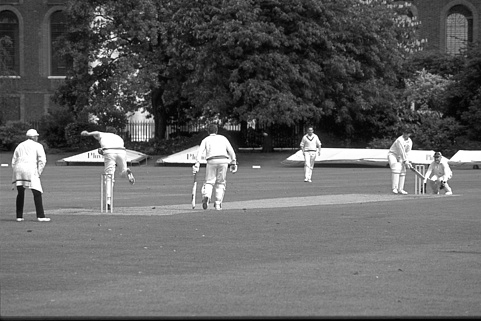

Supplement: Supplementary file 1 [file jimaging-11-00199-s001.zip › DWCA_code/dataset/color250/102_1.png]

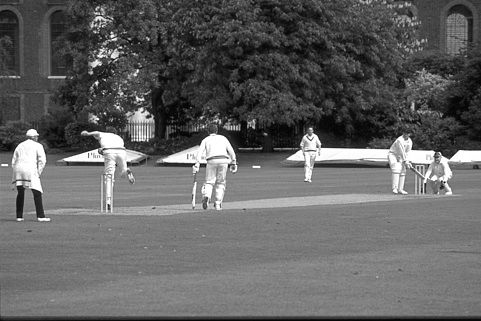

Supplement: Supplementary file 1 [file jimaging-11-00199-s001.zip › DWCA_code/dataset/color250/102_2.png]

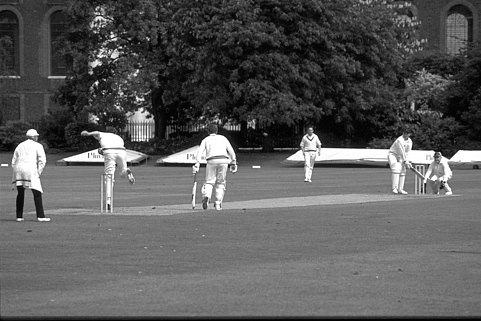

Supplement: Supplementary file 1 [file jimaging-11-00199-s001.zip › DWCA_code/dataset/color250/102_3.png]

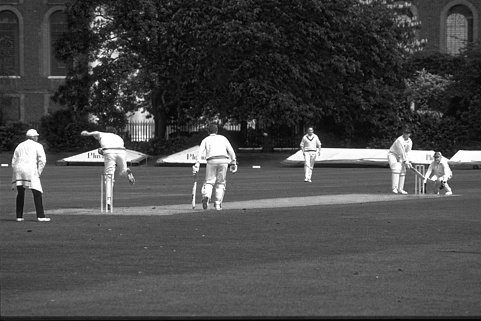

Supplement: Supplementary file 1 [file jimaging-11-00199-s001.zip › DWCA_code/dataset/color250/102_4.png]

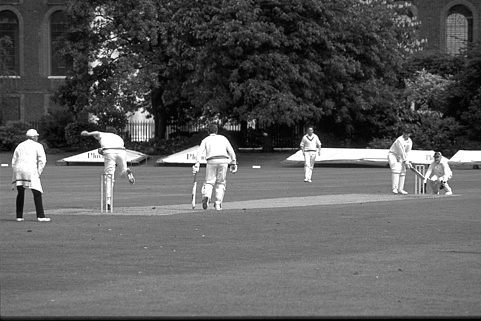

Supplement: Supplementary file 1 [file jimaging-11-00199-s001.zip › DWCA_code/dataset/color250/102_5.png]

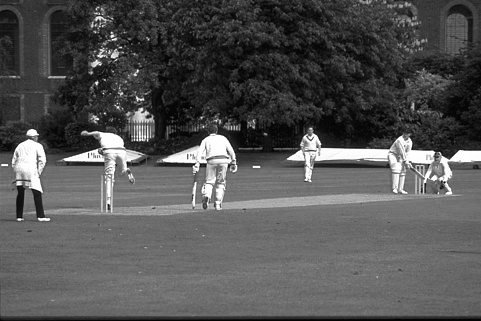

Supplement: Supplementary file 1 [file jimaging-11-00199-s001.zip › DWCA_code/dataset/color250/102_6.png]

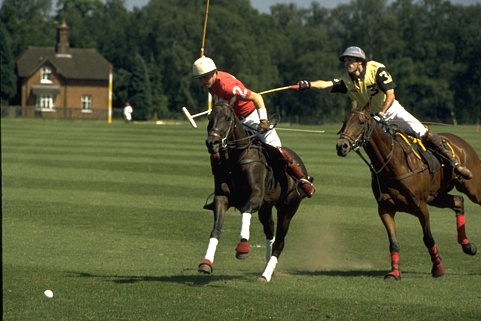

Supplement: Supplementary file 1 [file jimaging-11-00199-s001.zip › DWCA_code/dataset/color250/103.png]

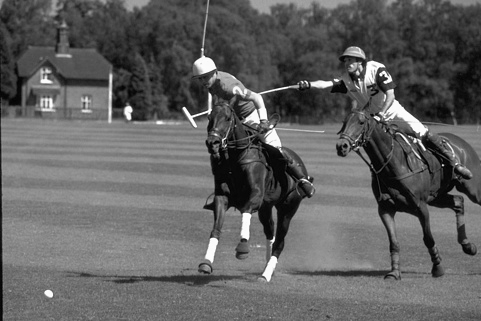

Supplement: Supplementary file 1 [file jimaging-11-00199-s001.zip › DWCA_code/dataset/color250/103_1.png]

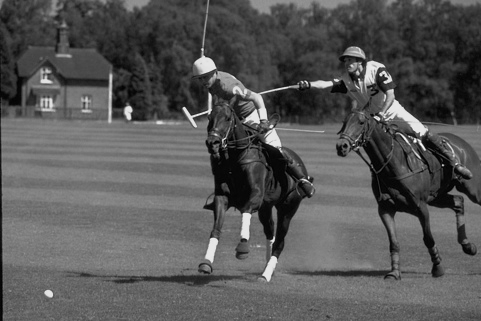

Supplement: Supplementary file 1 [file jimaging-11-00199-s001.zip › DWCA_code/dataset/color250/103_2.png]

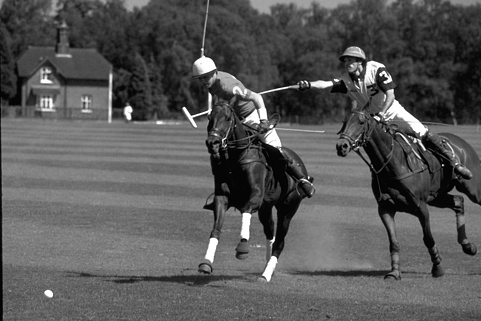

Supplement: Supplementary file 1 [file jimaging-11-00199-s001.zip › DWCA_code/dataset/color250/103_3.png]

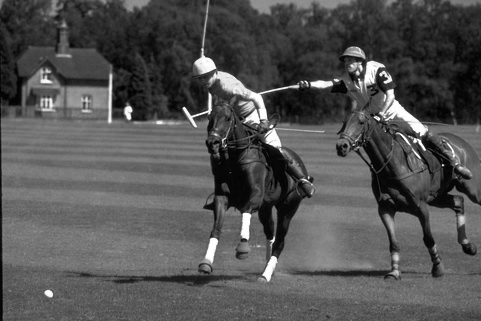

Supplement: Supplementary file 1 [file jimaging-11-00199-s001.zip › DWCA_code/dataset/color250/103_4.png]

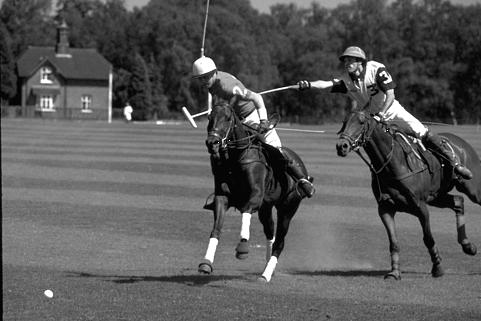

Supplement: Supplementary file 1 [file jimaging-11-00199-s001.zip › DWCA_code/dataset/color250/103_5.png]

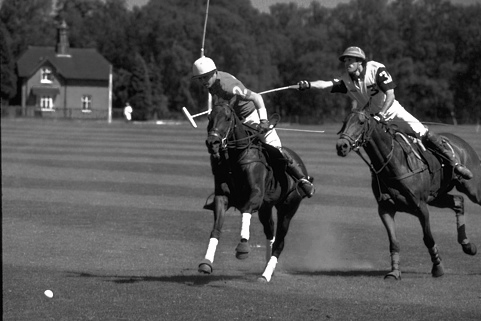

Supplement: Supplementary file 1 [file jimaging-11-00199-s001.zip › DWCA_code/dataset/color250/103_6.png]

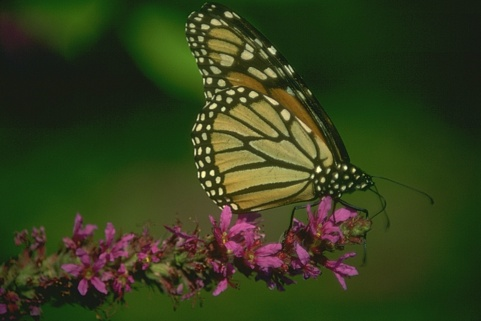

Supplement: Supplementary file 1 [file jimaging-11-00199-s001.zip › DWCA_code/dataset/color250/104.png]

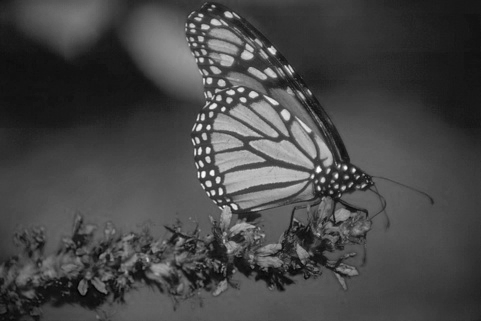

Supplement: Supplementary file 1 [file jimaging-11-00199-s001.zip › DWCA_code/dataset/color250/104_1.png]

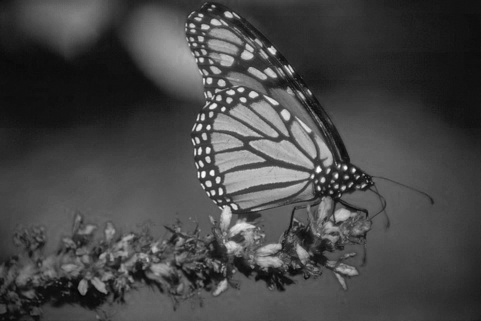

Supplement: Supplementary file 1 [file jimaging-11-00199-s001.zip › DWCA_code/dataset/color250/104_2.png]

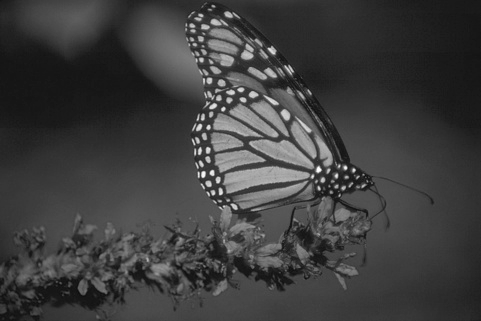

Supplement: Supplementary file 1 [file jimaging-11-00199-s001.zip › DWCA_code/dataset/color250/104_3.png]

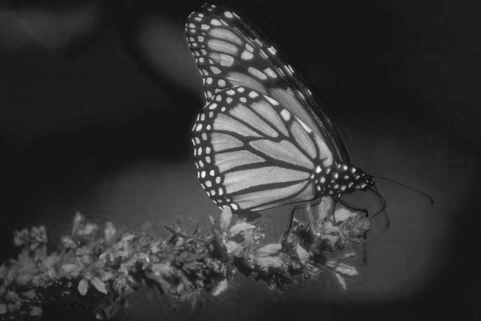

Supplement: Supplementary file 1 [file jimaging-11-00199-s001.zip › DWCA_code/dataset/color250/104_4.png]

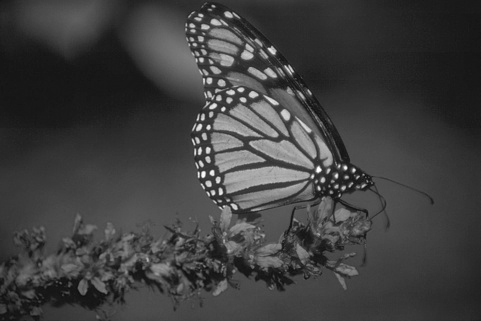

Supplement: Supplementary file 1 [file jimaging-11-00199-s001.zip › DWCA_code/dataset/color250/104_5.png]

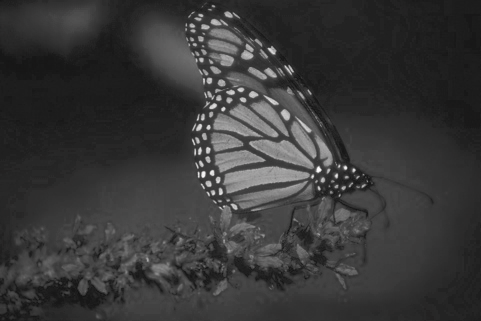

Supplement: Supplementary file 1 [file jimaging-11-00199-s001.zip › DWCA_code/dataset/color250/104_6.png]

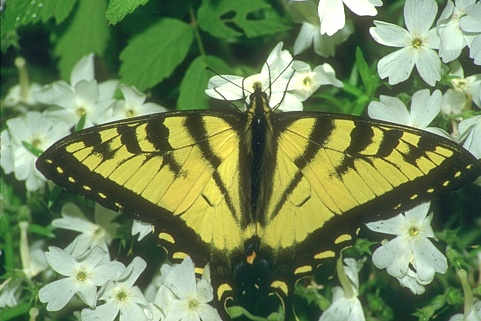

Supplement: Supplementary file 1 [file jimaging-11-00199-s001.zip › DWCA_code/dataset/color250/105.png]

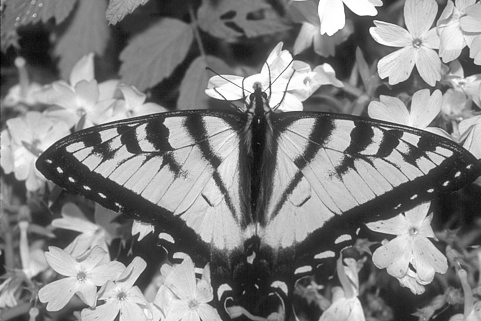

Supplement: Supplementary file 1 [file jimaging-11-00199-s001.zip › DWCA_code/dataset/color250/105_1.png]

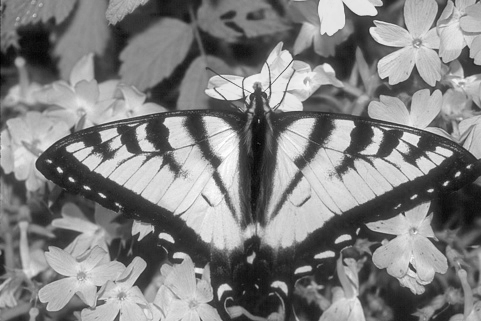

Supplement: Supplementary file 1 [file jimaging-11-00199-s001.zip › DWCA_code/dataset/color250/105_2.png]

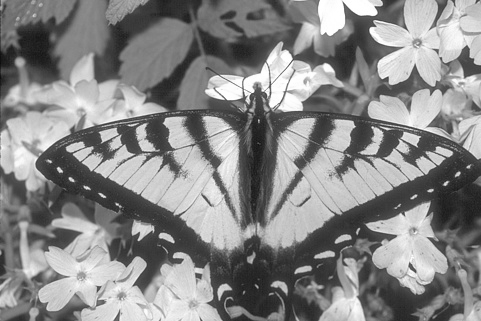

Supplement: Supplementary file 1 [file jimaging-11-00199-s001.zip › DWCA_code/dataset/color250/105_3.png]

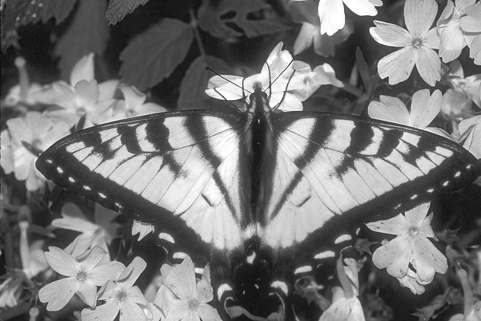

Supplement: Supplementary file 1 [file jimaging-11-00199-s001.zip › DWCA_code/dataset/color250/105_4.png]

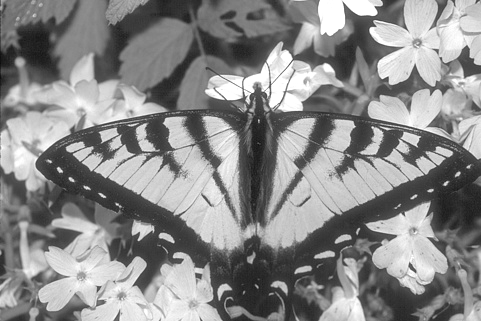

Supplement: Supplementary file 1 [file jimaging-11-00199-s001.zip › DWCA_code/dataset/color250/105_5.png]

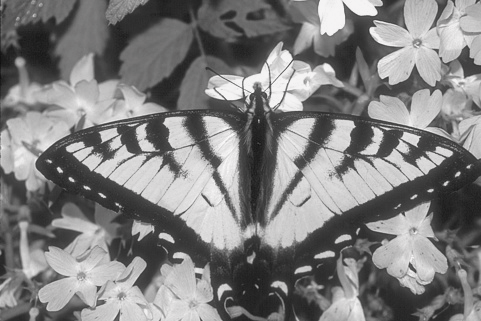

Supplement: Supplementary file 1 [file jimaging-11-00199-s001.zip › DWCA_code/dataset/color250/105_6.png]

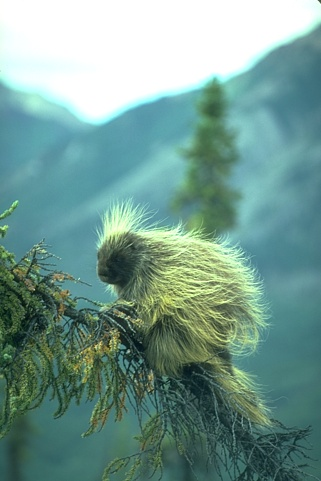

Supplement: Supplementary file 1 [file jimaging-11-00199-s001.zip › DWCA_code/dataset/color250/106.png]

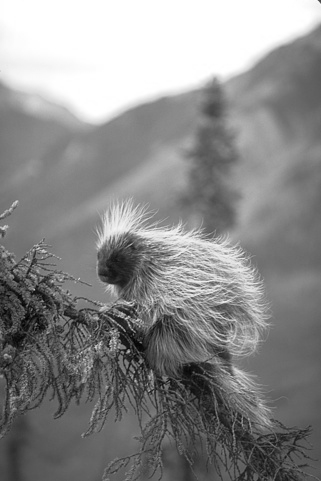

Supplement: Supplementary file 1 [file jimaging-11-00199-s001.zip › DWCA_code/dataset/color250/106_1.png]

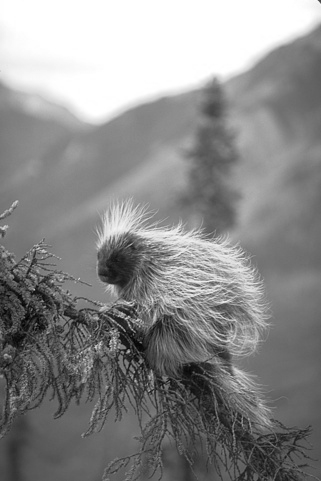

Supplement: Supplementary file 1 [file jimaging-11-00199-s001.zip › DWCA_code/dataset/color250/106_2.png]

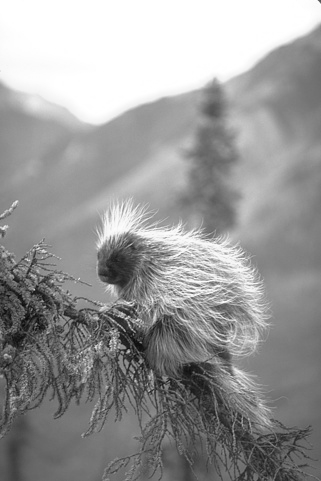

Supplement: Supplementary file 1 [file jimaging-11-00199-s001.zip › DWCA_code/dataset/color250/106_3.png]

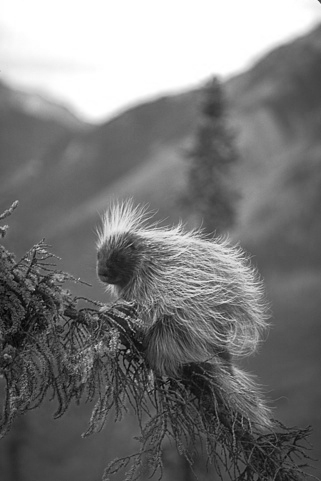

Supplement: Supplementary file 1 [file jimaging-11-00199-s001.zip › DWCA_code/dataset/color250/106_4.png]

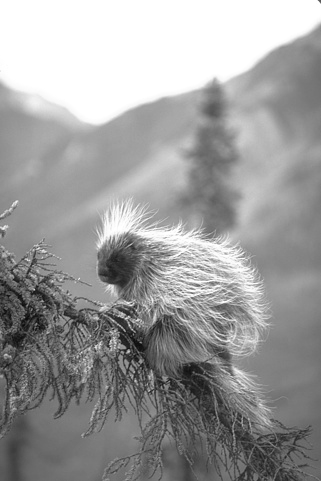

Supplement: Supplementary file 1 [file jimaging-11-00199-s001.zip › DWCA_code/dataset/color250/106_5.png]

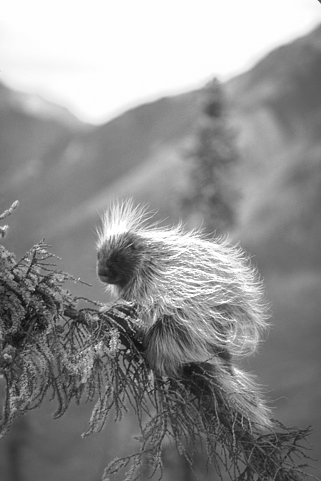

Supplement: Supplementary file 1 [file jimaging-11-00199-s001.zip › DWCA_code/dataset/color250/106_6.png]

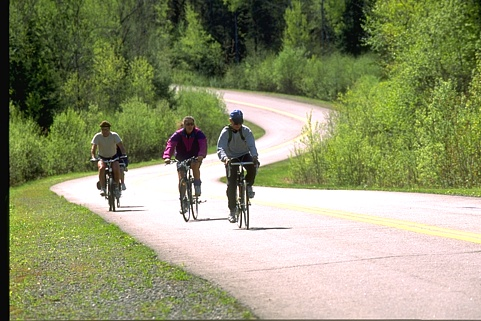

Supplement: Supplementary file 1 [file jimaging-11-00199-s001.zip › DWCA_code/dataset/color250/107.png]

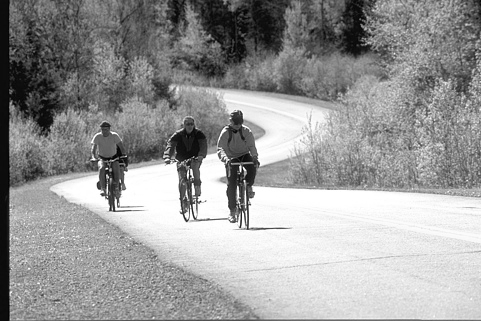

Supplement: Supplementary file 1 [file jimaging-11-00199-s001.zip › DWCA_code/dataset/color250/107_1.png]

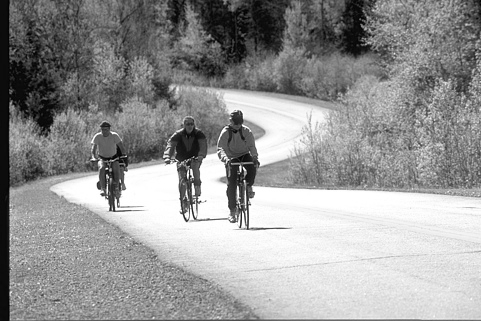

Supplement: Supplementary file 1 [file jimaging-11-00199-s001.zip › DWCA_code/dataset/color250/107_2.png]

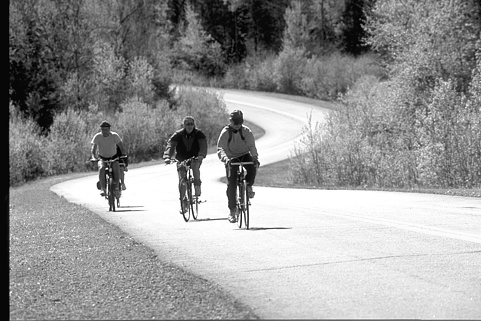

Supplement: Supplementary file 1 [file jimaging-11-00199-s001.zip › DWCA_code/dataset/color250/107_3.png]

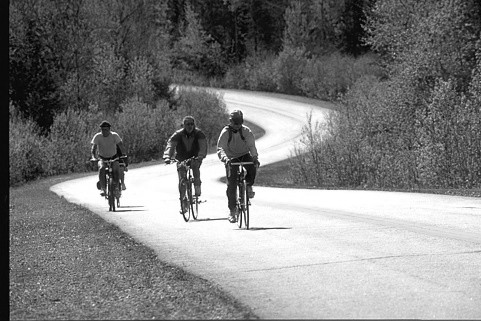

Supplement: Supplementary file 1 [file jimaging-11-00199-s001.zip › DWCA_code/dataset/color250/107_4.png]

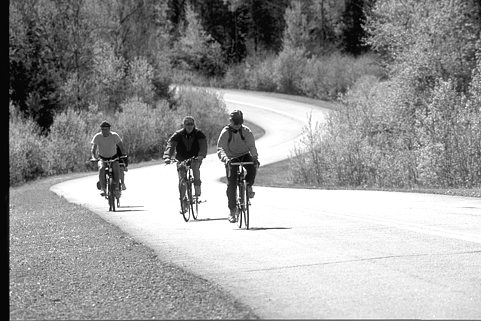

Supplement: Supplementary file 1 [file jimaging-11-00199-s001.zip › DWCA_code/dataset/color250/107_5.png]

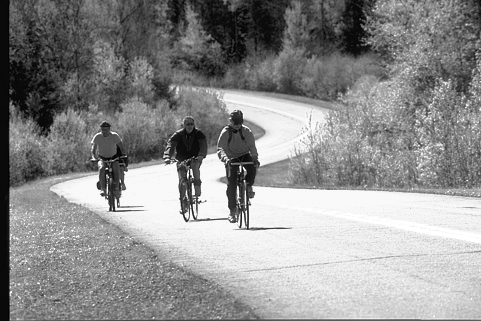

Supplement: Supplementary file 1 [file jimaging-11-00199-s001.zip › DWCA_code/dataset/color250/107_6.png]

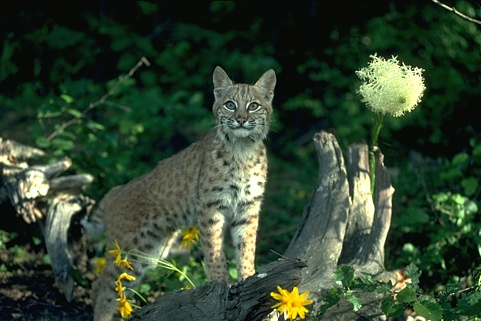

Supplement: Supplementary file 1 [file jimaging-11-00199-s001.zip › DWCA_code/dataset/color250/108.png]

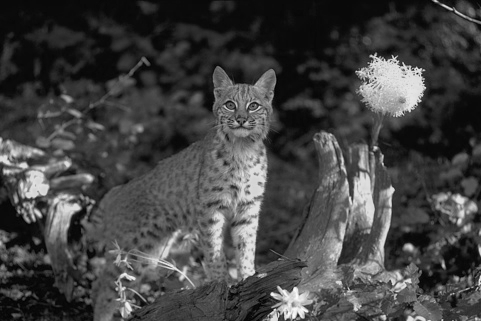

Supplement: Supplementary file 1 [file jimaging-11-00199-s001.zip › DWCA_code/dataset/color250/108_1.png]

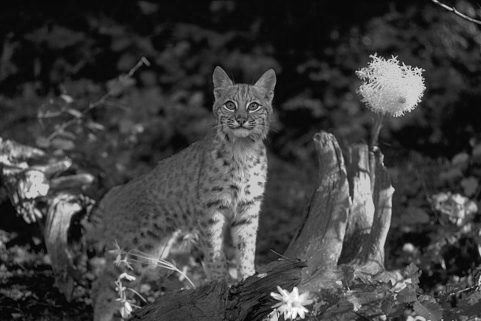

Supplement: Supplementary file 1 [file jimaging-11-00199-s001.zip › DWCA_code/dataset/color250/108_2.png]

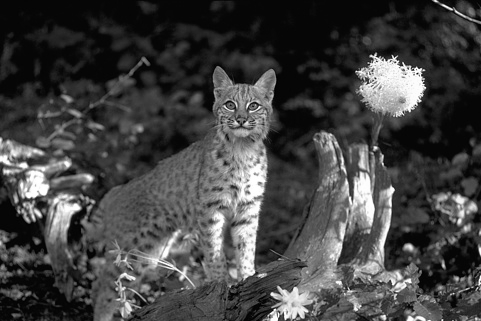

Supplement: Supplementary file 1 [file jimaging-11-00199-s001.zip › DWCA_code/dataset/color250/108_3.png]

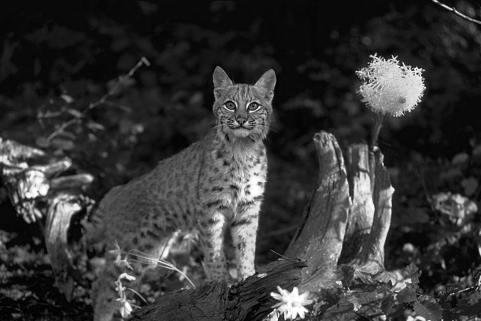

Supplement: Supplementary file 1 [file jimaging-11-00199-s001.zip › DWCA_code/dataset/color250/108_4.png]

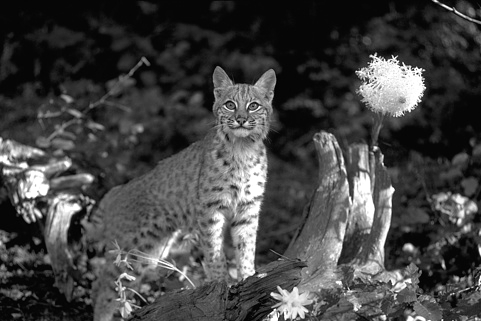

Supplement: Supplementary file 1 [file jimaging-11-00199-s001.zip › DWCA_code/dataset/color250/108_5.png]

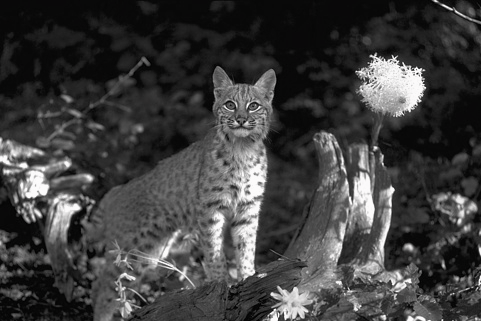

Supplement: Supplementary file 1 [file jimaging-11-00199-s001.zip › DWCA_code/dataset/color250/108_6.png]

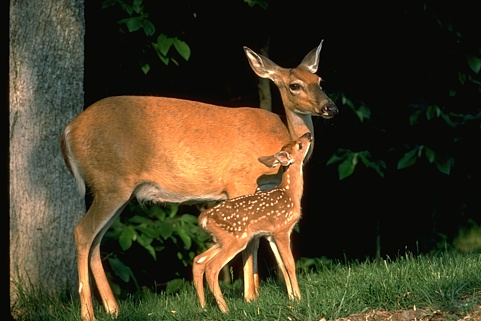

Supplement: Supplementary file 1 [file jimaging-11-00199-s001.zip › DWCA_code/dataset/color250/109.png]

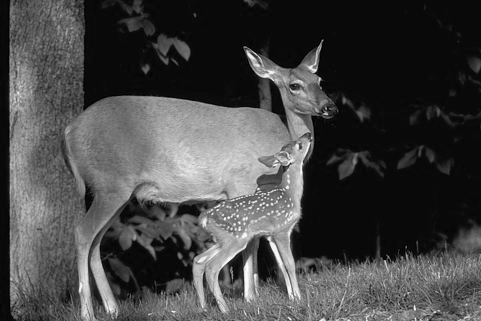

Supplement: Supplementary file 1 [file jimaging-11-00199-s001.zip › DWCA_code/dataset/color250/109_1.png]

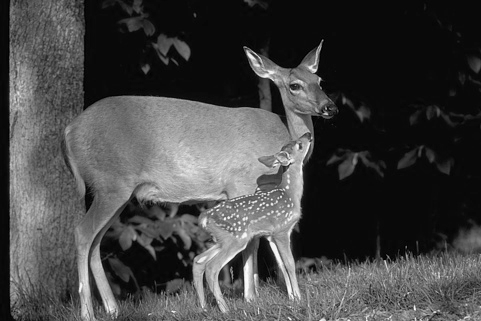

Supplement: Supplementary file 1 [file jimaging-11-00199-s001.zip › DWCA_code/dataset/color250/109_2.png]

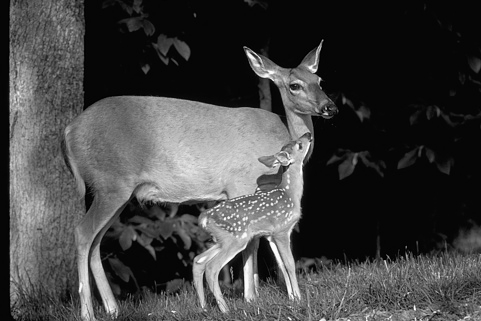

Supplement: Supplementary file 1 [file jimaging-11-00199-s001.zip › DWCA_code/dataset/color250/109_3.png]

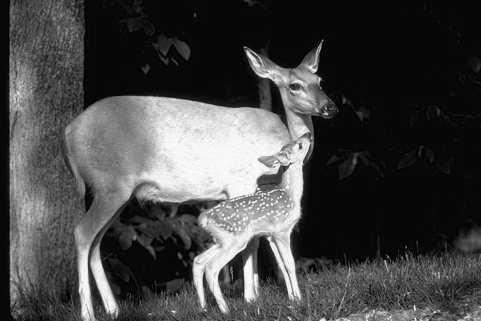

Supplement: Supplementary file 1 [file jimaging-11-00199-s001.zip › DWCA_code/dataset/color250/109_4.png]

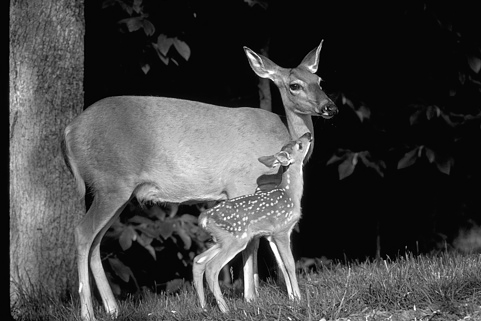

Supplement: Supplementary file 1 [file jimaging-11-00199-s001.zip › DWCA_code/dataset/color250/109_5.png]

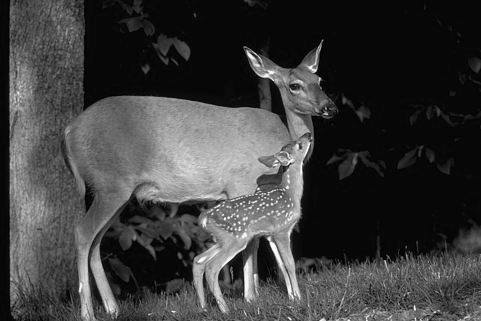

Supplement: Supplementary file 1 [file jimaging-11-00199-s001.zip › DWCA_code/dataset/color250/109_6.png]

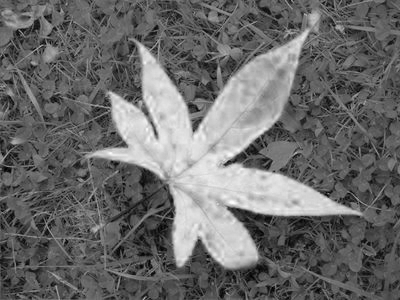

Supplement: Supplementary file 1 [file jimaging-11-00199-s001.zip › DWCA_code/dataset/color250/10_1.png]

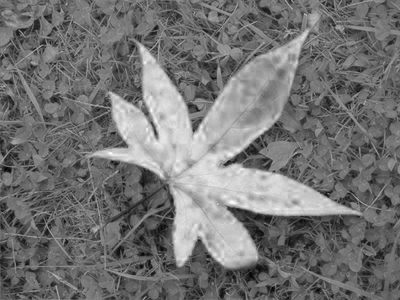

Supplement: Supplementary file 1 [file jimaging-11-00199-s001.zip › DWCA_code/dataset/color250/10_2.png]

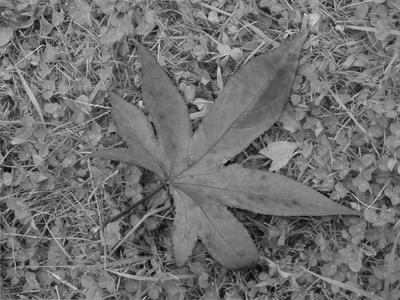

Supplement: Supplementary file 1 [file jimaging-11-00199-s001.zip › DWCA_code/dataset/color250/10_3.png]

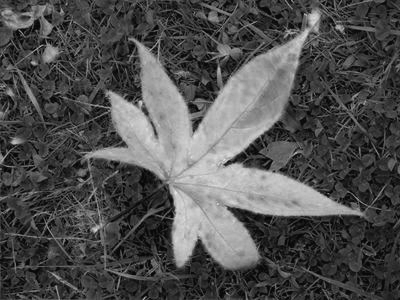

Supplement: Supplementary file 1 [file jimaging-11-00199-s001.zip › DWCA_code/dataset/color250/10_4.png]
